# Real-time context-dependent cooperation in parental provisioning reveals fitness payoffs in barn owls

**DOI:** 10.1016/j.isci.2025.113533

**Published:** 2025-09-10

**Authors:** Paolo Becciu, Kim Schalcher, Estelle Milliet, James L. Savage, Andrea Romano, Bettina Almasi, Alexandre Roulin

**Affiliations:** 1 Department of Ecology and Evolution, University of Lausanne, 1015 Lausanne, Switzerland; 2 Southern Institute of Technology, Invercargill 9840, New Zealand; 3 Department of Environmental Science and Policy, University of Milan, 20122 Milan, Italy; 4 Swiss Ornithological Institute, 6204 Sempach, Switzerland

**Keywords:** Ecology, Zoology, Ornithology

## Abstract

Parental cooperation in species with extended biparental care is essential for offspring survival, yet real-time negotiation and coordination in the wild remain poorly understood. We simultaneously Global Positioning System (GPS)- and accelerometer-tracked 68 breeding pairs of barn owls (*Tyto alba*) during chick rearing, quantifying parents’ hunting effort, prey deliveries, self-feeding, nest attendance, and partner encounters. Within pairs, parental investment was highly plastic, with low repeatability of nightly provisioning shares. Females increased provisioning when males underperformed or when foraging habitat was likely poor. Parents synchronized foraging schedules and nest visits, exhibiting turn-taking-like coordination; pairs that shared provisioning more equally foraged in parallel overnight and met frequently at the nest. We detected sequential, between-night adjustments, whereby effort on one night influenced provisioning the next. Pairs maintaining more equitable care achieved higher survival and growth in their later-hatching nestlings. Our findings demonstrate how high-resolution biologging reveals dynamic behavioral mechanisms underpinning flexible biparental care under ecological variability.

## Highlights

• *High-resolution GPS-accelerometer biologging concurrently tracks both barn owl parents*
• Females partially compensate when males’ hunting performance or condition declines
• Coordinated pairs with frequent and brief nest meetings share more equal provisioning
• *Equitable provisioning enhances nestling survival, and growth of late-hatched chicks*

## Graphical abstract

**Figure:**
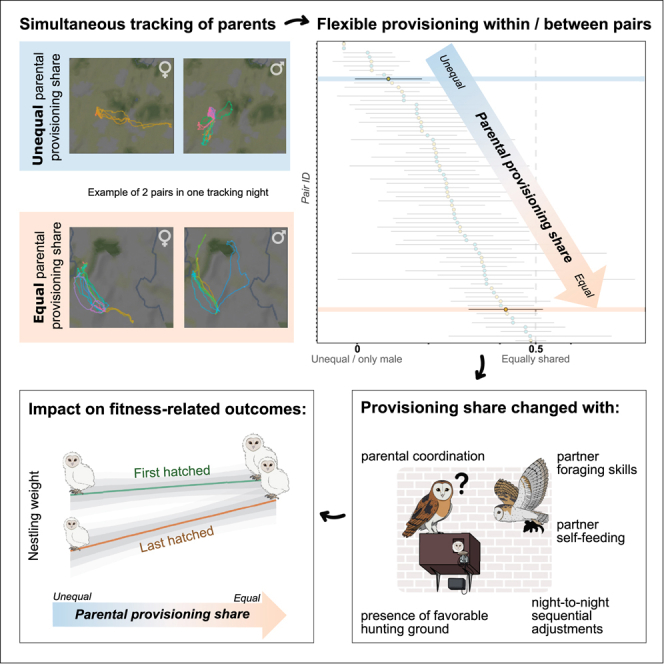

## Introduction

Extended biparental care represents one of the most striking examples of cooperative behavior in animals, yet it simultaneously embodies a fundamental conflict: each parent’s fitness increases if its partner bears more of the costs of care.^1^^,^^2^ In avian systems, these costs can include energy expenditure on foraging, increased predation risk, less time for self-maintenance, lost future reproductive opportunities, and poorer survival.^1^^,^^3^^,^^4^^,^^5^^,^^6^ Game-theoretic models—both sealed-bid frameworks and negotiation models—predict the outcome of this conflict over care to be partial compensation by each parent when the other’s effort changes,^7^^,^^8^ where parents adjust their provisioning dynamically.^8^^,^^9^ Empirical tests have generally supported that, when one parent is handicapped or experimentally deterred, its partner typically increases investment, but that total provisioning remains lower.^4^^,^^10^^,^^11^^,^^12^ This body of theory and evidence suggests that the outcome of conflicts over care is rarely optimal for either sex; instead, parents balance their investment to minimize brood failure without risking partner desertion.^6^^,^^13^ Under this perspective, parental investment is shaped not only by immediate brood demands but also by the history of interactions between mates.^5^^,^^14^

Sex-specific role specialization and state-dependent constraints can further influence parental strategies. Many species require multiple types of care (e.g., feeding, brooding, defense), and males and females often perform different roles. Social role specialization—each parent focusing on distinct aspects of care—can maximize joint fitness when both tasks incur synergistic costs.^15^ Under high brood demand or low resource availability, specialization is predicted to give way to more evenly shared provisioning, as both parents must contribute substantially. Under opposite conditions, more pronounced task division yields efficiency gains. This framework explains why many raptors show sex-specific roles (e.g., males hunting more, females brooding more) yet dynamically adjust those roles as brood needs and environmental conditions shift.^16^

Individual condition and habitat quality modulate these sex-specific role patterns. Each parent’s energy reserves, body condition, and environmental context (e.g., prey availability, habitat quality) determine optimal investment at any given moment.^17^^,^^18^^,^^19^ When in a positive state (good condition, rich habitat), the future cost of investing extra effort is low, so care increases; conversely in negative states parents conserve energy for self-maintenance, reducing care.^17^^,^^20^^,^^21^ Also, an increase in one parent’s hunting success should reduce the marginal benefit of the other’s effort, leading to an indirect suppression of the partner’s provisioning.^6^^,^^22^ Such state- and condition-dependent adjustments are thought to underlie flexible provisioning rules.^22^ They also set the stage for dynamic feedback between parents: the effort one parent expends affects its future state, which can in turn alter how the pair divides work moving forward.^9^^,^^23^

Sequential adjustments and temporal negotiation shape overall investment patterns. Theoretical extensions of negotiation models to repeated interactions^24^^,^^25^ predict that a heavy investment in date *n* depletes reserves and lowers investment in date *n + 1*, unless the partner compensates. Over the course of offspring rearing, this can lead to alternating high- and low-effort days, or to a gradual drift in provisioning levels. In long-lived seabirds, for example, coordination levels can change across breeding phases and even carry over from incubation to chick-rearing.^5^^,^^26^ Such findings align with the idea that parents use rules-of-thumb for negotiation (e.g., alternating duties or maintaining overall parity) that smooth out short-term fluctuations.^27^ However, direct evidence of sequential adjustments on a day-to-day basis in wild animals remains scarce, mostly due to limitations in monitoring both parents continuously.

Finally, temporal coordination via the timing and frequency of nest encounters or nest-relief events represents a more subtle dimension of cooperation. Theoretical models of turn-taking show that conditional cooperation—where each parent is more likely to provision if the other has done so recently—emerges as an evolutionarily stable strategy^24^ even under imperfect monitoring or differences between pairs.^27^ In nocturnal species parents may rely on intrinsic schedules or indirect cues (e.g., offspring vocalizations) to coordinate, but encounter rate at the nest and nest attendance duration should influence subsequent provisioning as frequent encounters allow direct assessment of partner contribution and condition, triggering strategic adjustments.^28^

Despite rich theoretical predictions, tests of these models under natural conditions remain piecemeal, largely because fine-scale measurement of individual parental behavior away from the nest remains challenging. It is still unclear how each parent’s foraging behavior and success influence their own provisioning contributions and, consequently, their partner’s level of cooperation in offspring rearing.^28^ In socially monogamous species, the division of parental duties is rarely fixed, often shifting in response to both social dynamics and environmental variability.^5^^,^^9^^,^^16^^,^^22^^,^^29^ New technologies, such as the combination of Global Positioning Systems (GPSs) and triaxial accelerometers, can help us collect refined behavioral data about individuals^30^^,^^31^^,^^32^ potentially highlighting short-term responses, and simultaneous tracking of parents can provide invaluable insights on cooperation in biparental care systems.^26^ Simultaneous tracking can be a powerful tool to investigate the parental response to social and environmental conditions that parents face when raising offspring.

Here, we leverage simultaneous GPS and accelerometer biologging deployed on free-living barn owl (*Tyto alba*) pairs to continuously track foraging trips, hunting success, prey deliveries, self-feeding rate, nest attendance, and interparental encounters over consecutive nights. Barn owls forage at night over fairly large areas, making them an ideal system to test six explicit hypotheses derived from conflict, negotiation, specialization, state-dependence, and temporal-coordination theories:

H 1—partial compensation hypothesis: parents will show partial compensation in provisioning following partner effort changes,^8^ and parental provisioning will vary with prey availability—under poorer conditions pairs should share provisioning investment more equitably, whereas in rich environments a single parent (mainly the father in barn owls) may successfully maintain brood provisioning alone^22^^,^^33^;

H 2—sex-specific role specialization hypothesis: sex-specific provisioning biases will shift when brood demand or partner performance varies,^15^^,^^16^ in barn owls the male acts as the primary food provider while the female spends more time at the nest (or prospecting^34^), yielding a consistently male-biased provisioning share;

H 3—state-dependent investment hypothesis: individual body condition and foraging success will correlate positively with care, reflecting dynamic cost-benefit decisions,^17^^,^^20^ parents with low self-feeding rates and higher hunting performance will provision more, whereas energetically stressed parents will prioritize self-feeding;

H 4—coordinated foraging hypothesis: in pairs with more equitable provisioning, females will forage concurrently with males throughout the night and will exhibit higher nest-encounter rates. In contrast, in pairs with unbalanced provisioning, females will cease foraging earlier and show fewer nest meetings^24^^,^^28^;

H 5—sequential adjustment hypothesis: a lower female provisioning share on one night will influence subsequent nights through both parental state and offspring demand, indicating short-term compensatory adjustments across successive nights^24^^,^^25^;

H 6—offspring fitness outcome hypothesis: more equitable provisioning and higher total parental investment will enhance offspring growth and survival.^24^

By integrating theoretical frameworks with high-resolution movement and behavioral data, our findings provide insights into the mechanisms underlying biparental care flexibility in barn owls, offering a template for assessing complex parental strategies in other taxa and providing a real-time examination of how two parents balance conflict and cooperation to optimize offspring provisioning under natural ecological constraints.^24^^,^^25^ These insights contribute to broader discussions on the evolution of parental care systems and the resilience of these systems in the face of environmental changes.

## Results

We analyzed the hunting and provisioning behavior of 136 barn owls (68 breeding pairs) over 333 nights, using GPS and accelerometer data (mean ± SD: 5.23 ± 0.52 nights per pair, range: 4–6 nights). Across all pairs, we recorded 20,553 hunting attempts—13,746 by males and 6,807 by females—with males achieving a mean success rate of 31 ± 12% and females achieving 27 ± 19% . Males undertook 3,089 foraging trips (mean 8.69 trips per night, range: 1–31), whereas females made 1,192 trips (mean 3.35 trips per night, range: 0–14). Additional summarized information regarding sex and pair variables can be found in Table S1. For each model the samples size slightly changed given missing data for specific nights and individuals across variables (refer to specific model tables in STAR Methods).

### Variation in biparental provisioning (H1 and 2)

**Figure 1:**
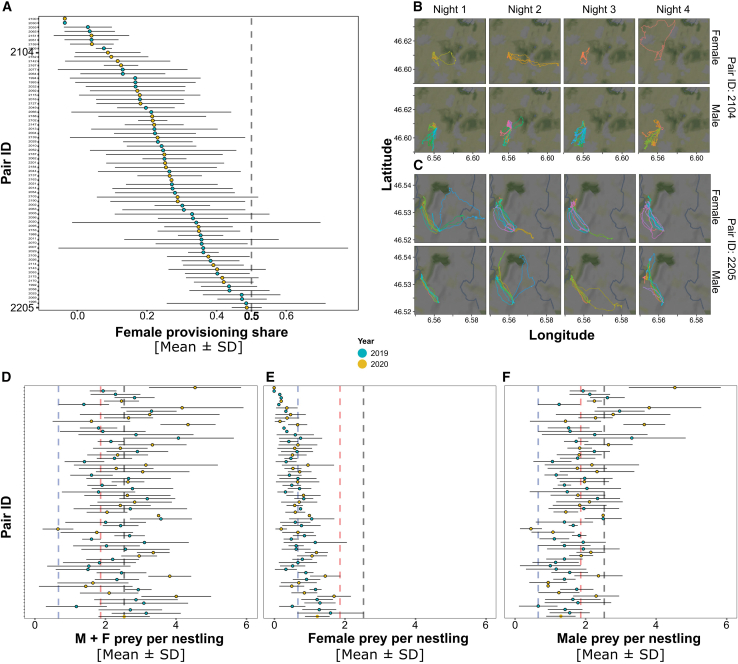
Within-pair variation in provisioning ratio and relative parental nestling provisioning Summary values per pair of (A) mean and variation of female provisioning share, measurement used to evaluate the balance in prey deliveries within the pair and specifically is the proportion of prey brought back to the nest by the female relative to the total brought by both parents, the dashed line highlights the equal investment from male and female parent; (B) example of female (top row) and male (bottom row) tracks foraging for the brood in a pair with male contributing the most to the chick provisioning (different colors are different foraging trips); (C) example of female (top row) and male (bottom row) tracks foraging for the brood in a pair with parents contributing almost equally to the chick provisioning (different colors are different foraging trips); (D) mean and variation of prey items per nestling by both parents combined; (E) mean and variation of prey per nestling by female parent; (F) mean and variation of prey per nestling by male parent. Dots are mean values; the range is the standard deviation. Dots are colored by year, and they are arranged from top (low female contribution to high male contribution) to bottom (more equitable pairs) by mean female provisioning share, as displayed in A. Vertical dashed lines are the overall mean of prey items per nestling by both parents (black), by male parent (red), and by female parent (blue). Data are represented as mean ± SD.

Parental share in chick provisioning varied widely among pairs, from exclusive male provisioning to equal sharing (variation in female provisioning share, as shown in Figures 1A–1C). The mean female provisioning share (proportion of prey deliveries by the female) was 0.27 ± 0.17 (hence male ratio 0.73; Figure 1A). Low repeatability of female provisioning share across nights (R = 0.115, 95% C.I. = [0.06, 0.15]; controlled for year and brood size) indicates within-pair flexibility. On average, females delivered 0.67 ± 0.52 prey items per nestling per night and males 1.86 ± 0.98 for a combined 2.53 ± 1.18 prey items per nestling per night (Figures 1D–1F). Provisioning balance tended to increase with brood size (Figure S2). Pairs with three or fewer nestlings showed lower female provisioning shares than those with five nestlings (estimate: −0.74, highest posterior density [HPD]: −1.40 to −0.08), while five-nestling broods had higher ratios than six or more (estimate: 0.98, HPD: 0.14 to 1.87; Figure S2; Table S2).

### Within-night adjustments of parental effort (H1 to 4)

Nightly female provisioning share correlated positively with their own prey-per-nestling rate (ρ = 0.66, 95% credible interval [Cr.I.] = [0.60, 0.72], iterations = 10,000, Figure S3) and negatively with the male’s provisioning rate (ρ = −0.39, 95% Cr.I. = [-0.47, −0.29], iterations = 10,000, Figure S4), indicating that increased female effort promotes a more equitable division of parental care (chick provisioning). These patterns remained consistent when averaged per individual (Figures S5 and S6).

**Figure 2:**
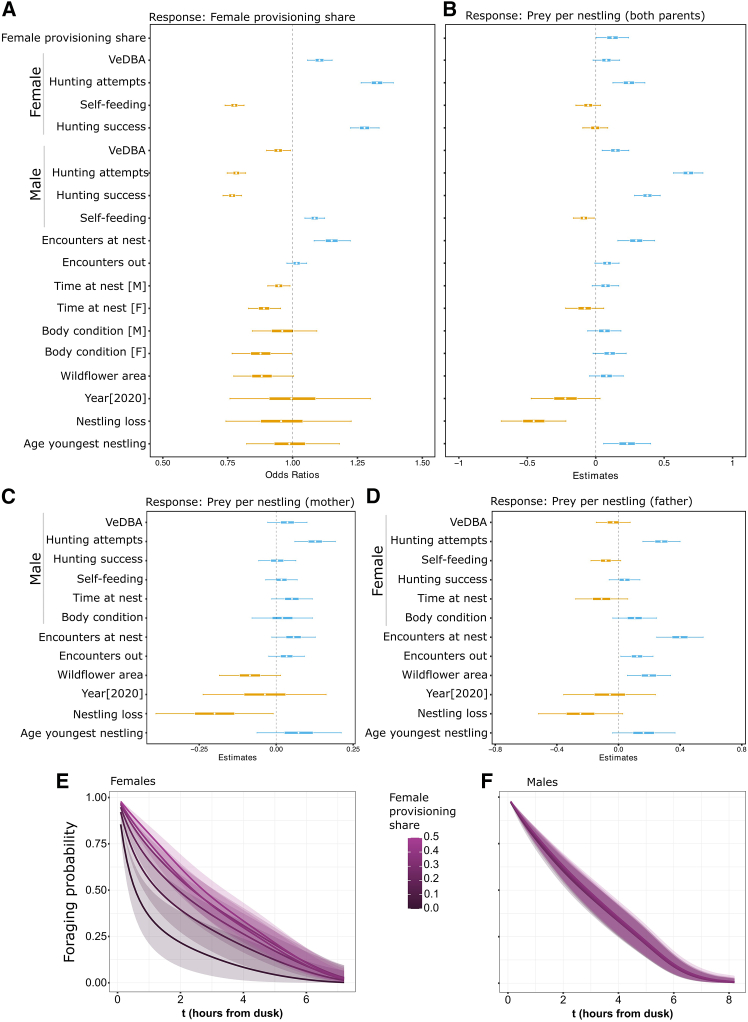
Predictors of flexible chick provisioning and temporal dynamics of foraging probability Summary plots of the Bayesian Generalized and Linear mixed effect models showing the standardized effects of foraging performance and behaviors of parents, area of wildflower strips, year, and brood size on female provisioning share (A), prey per nestling delivered by both parents (B), on relative chick provisioning by female (C) and by male (D) (see Tables S3-S6). Standardized effects are expressed as posterior distributions (horizontal boxplots) with a mean (white dot), the 50% Credible Intervals (the box), and the 95% Credible Intervals limits (the whiskers). The categorical variables “Year” and “Nestling loss” show the result of comparison between the group shown in squared brackets, [2020] and [1], and their reference level, [2019] and [0], respectively. The bottom panel shows the output of the time-to-event Piece-wise Exponential Additive Mixed Model (PAMM) as the probability of foraging (*y* axis) predicted at any time of the night (*x* axis), expressed as hours from sunset, depending on female provisioning share proportion (purple-colored lines and shaded 95% Confidence Intervals) for females (E) and males (F).

We modeled female provisioning share as a function of both parents’ foraging behavior (attempts, success, Vectorial Dynamic Body Acceleration [VeDBA]), self-feeding rate, encounters at and outside the nest, time spent at the nest, body condition at tagging (Scaled Mass Index [SMI]), year, wildflower strip area (as preferred hunting habitat,^35^^,^^36^^,^^37^; Table S3), nestling mortality during tagging period, and age of youngest nestling (Table S4). A one-SD increase in wildflower strip area was associated with a 12% decrease in female provisioning (95% Cr.I. = [-22.7%, 0.1%]), while one-SD increase in male hunting attempts, success rate, and VeDBA corresponded to decreases in female provisioning of 21.7% (95% Cr.I. = [-25.2%, −18.0%]), 23% (95% Cr.I. = [-26.8%, −19.6%]), and 5.6% (95% Cr.I. = [-10.0%, −0.9%]), respectively. In contrast, each one-SD rise in male self-feeding resulted in an 8% increase in female provisioning (95% Cr.I. = [4.7%, 12.3%]; Figure 2A; Table S4). Female provisioning share increased with her own hunting performance—attempts, 33% (95% Cr.I. = [26.5%, 38.8%]); success rate, 28% (95% Cr.I. = [22.3%, 33.4%]); and VeDBA, 10% (95% Cr.I. = [5.7%, 15.2%])—and declined with her own self-feeding (−22.4%, 95% Cr.I. = [-26.0%, −18.8%]; Figure 2A; Table S4). More frequent nest encounters also boosted female provisioning (15% per one-SD, 95% Cr.I. = [8.3%, 22.3%]), whereas longer nest attendance by either parent reduced it (male: −5%, 95% Cr.I. = [-9.6%, −1.0%]; female: 11%, 95% Cr.I. = [-17.0%, −4.7%]). Female body condition at tagging was negatively related (−12% per SD, 95% Cr.I. = [-23.3%, −0.6%]), but male condition was not (Figure 2A; Table S4). Neither nestling loss nor nestling age nor tagging year had a substantial effect.

Pairs that shared provisioning more equally delivered more prey per nestling per night. Total prey per nestling increased with female provisioning share (Figure 2B), with male hunting attempts exerting the strongest positive effect, followed by male success and female hunting attempts; male self-feeding had a negative effect (median of the posterior distribution [MPD] = −0.09, 95% Cr.I. = [-0.16, −0.01]). Nest encounters were also positively related to total provisioning (MPD = 0.30, 95% Cr.I. = [0.16, 0.43]). Habitat, year, and most behavioral factors showed weak or non-meaningful effects, although nestling mortality reduced provisioning and nestling age had a modest positive effect (Table S5).

Reciprocal effects emerged when modeling individual prey delivery: female prey per nestling increased with male hunting attempts, weakly with nest encounters as well as time spent at the nest by the male, and declined with nestling loss and weakly with wildflower strip area (Figure 2C; Table S6); male prey per nestling increased with female hunting attempts, both nest and non-nest encounters, and wildflower strip area (Figure 2D; Table S7).

We estimated the probability of continued male and female foraging, defined as the likelihood of initiating a foraging trip as a function of time during the night (see STAR Methods), using a piece-wise exponential additive mixed model (PAMM) with smooth terms for time since dusk, female provisioning share, and their interaction. Male foraging probability declined steadily over the night in a near-uniform fashion across all levels of female provisioning share (effective degrees of freedom [edf] = 2.68, *p* = 0.64), indicating that male foraging trips were largely insensitive to the female’s contribution to the total prey delivered (Figure 2F). In females, foraging probability declined non-linearly over the night (edf = 3.62, *p* < 0.001). Crucially, this decline was steeper when females bore a larger share of provisioning: in pairs with low female provisioning share, females reduced their foraging probability by roughly 75% within the first 2 h after dusk, whereas in pairs with near-equal provisioning (higher female provisioning share), the female foraging curve closely tracked that of males (Figure 2E). This interaction between time and provisioning share (edf = 3.59, *p* < 0.001) underscores behavioral flexibility in females and highlights the trade-off between time, energy expenditure, and increased foraging effort as female provisioning share rises.

### Between-night sequential adjustments (H5)

To examine whether parental effort and foraging outcomes on one night influence behavior on the following night, we fitted models predicting three response variables—female provisioning share, total prey per nestling (both parents), prey per nestling by the mother, and prey per nestling by the father—using previous-night values of those same metrics alongside a suite of covariates. These covariates included each parent’s prior-night hunting attempts, hunting success, VeDBA, self-feeding rate, encounters at and outside the nest, time spent at the nest, wildflower strip area, nestling mortality during the tagging period, age of the youngest nestling, and tagging year. A one-SD increase in female provisioning share on the previous night predicted a 61% decrease of her provisioning ratio on the focal night (95% Cr.I. = [-68.2%, −52.7%]), indicating a partial compensatory adjustment (Table S8). Similarly, one-SD increase in male VeDBA, hunting success, and self-feeding on night *n – 1* were each associated with reduced female provisioning on night *n* by 11% (95% Cr.I. = [-16.1%, −5.9%]), 5.5% (95% Cr.I. = [-10.4%, −0.5%]) and 6% (95% Cr.I. = [-9.6%, −1.9%]), respectively, whereas more frequent (one-SD increase) nest encounters between parents on the previous night led to increased female contribution by 14% (95% Cr.I. = 5.9%, 22.7%]). In contrast, one-SD increase in nest attendance by both sexes and encounters away from the nest on the previous night corresponded to a 14% and 6% decrease, respectively, of female provisioning share on the subsequent night (Table S8; Figure S8).

Prey per nestling delivered by both parents exhibited negative relationship with previous-night values (MPD = −0.46, 95% Cr.I. = [–0.70, −0.22]). However, high female hunting attempts on the prior night led to higher total provisioning per nestling on the focal night (MPD = 0.26, 95% Cr.I. = [0.02, 0.51]). Other covariates showed no consistent effect on sequential adjustments of combined provisioning (Table S9; Figure S9).

When considering parental contributions separately, female prey delivery per nestling showed dependence on her own (MPD = −0.09, 95% Cr.I. = [-0.16, −0.02]) but not her partner’s previous-night behavior (Table S10; Figure S10). Similarly, male prey delivery per nestling was negatively related to its previous-night values (MPD = −0.16, 95% Cr.I. = [-0.29, −0.03]), but was not influenced by any previous-night female behaviors or encounters (Table S11; Figure S11).

### Contributions of specific parental behaviors to nestling survival and growth (H6)

We further analyzed nestling survival from hatching to logger recovery, incorporating individual parental behaviors (mean female provisioning share, combined and single parental provisioning per nestling), year, and wildflower strip area (see Figure S12; Table S12 in supplemental information). Mean female provisioning share showed a weak positive trend (MPD = 0.17, 95% Cr.I. = [-0.08, 0.44]), as did the combined prey delivery by both parents (MPD = 0.17, 95% Cr.I. = [-0.08, 0.44]). When analyzing single contributions by parent, female provisioning had a positive effect on nestling survival (MPD = 0.26, 95% Cr.I. = [0.01, 0.52]), whereas male provisioning showed no significant effect (MPD = 0.04, 95% Cr.I. = [-0.22, 0.31]). Additionally, male hunting attempts positively influenced nestling survival (MPD = 0.32, 95% Cr.I. = [0.04, 0.61]), while male hunting success rate had a negative effect (MPD = −0.24, 95% Cr.I. = [-0.49, −0.01]) and female success rate showed a positive trend (MPD = 0.23, 95% Cr.I. = [-0.02, 0.50]). Mean VeDBA, an energy expenditure proxy, was positively related to nestling survival for females (MPD = 0.25, 95% Cr.I. = [0.02, 0.49]) and showed a weaker association for males (MPD = 0.20, 95% Cr.I. = [-0.04, 0.44]).

**Figure 3:**
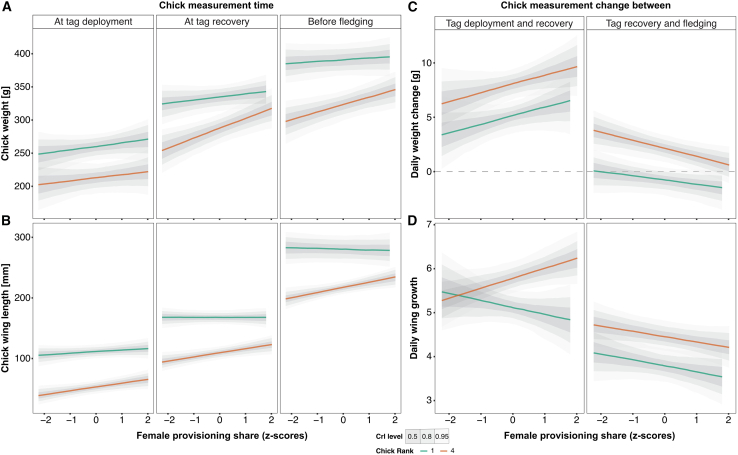
Relationships between mean female provisioning share and nestling growth Linear relationships between proxies of nestling growth (*y* axes in A, B, C, and D) and standardized values of mean female provisioning share (*x* axes) in interaction with the nestling rank (1 = oldest and 4 = born fourth or younger) divided per period of measurement (at tag deployment, tag recovery, and before fledging). Dashed lines represent daily growth equal 0. For visualization purposes we show only the relationship in the first (green) and last nestling rank (orange). Lines are the posterior means, and shaded areas are the predicted 50%, 80%, and 95% Credible Intervals. See also Tables S13–S24.

We assessed the effect of female provisioning share on nestling body condition by analyzing, in two separate models, nestling weight and wing length as a function of female provisioning share, measurement time (tag deployment, tag recovery, and pre-fledging), and nestling rank (4-level factor: 1 = born first, 2 = born second, 3 = born third, and 4 = born fourth or younger). We focused our analyses on the interaction terms between female provisioning share with measurement time and nestling rank, and on the slope differences (Figures 3A and 3B). Nestlings’ weight was overall weakly positively related to female provisioning share (MPD = 5.79, 95% Cr.I. = [0.00, 11.50]; Table S13). Specifically, female provisioning share proportion had the strongest relationship with the youngest (rank 4) nestlings’ weight at tag recovery (MPD = 15.15, 95% Cr.I. = [4.35, 26.4]) and before fledging (MPD = 11.44, 95% Cr.I. = [2.29, 20.7]), but not before tag deployment (Figure 3A; Tables S14 and S15). Wing length was also positively related to female provisioning share in the youngest nestlings for all three measurement times (Figure 3B; Tables S16–S18).

Daily weight gain also positively correlated with female provisioning share for all nestling ranks (Figure 3C; Tables S19–S21), while wing length growth increased with female provisioning share in younger nestlings but remained stable or slightly negative for the oldest (Figure 3D; Tables S22–S24) in the period between tag deployment and recovery. In the following period, between tag recovery and fledging, wing and weight change were flat or slightly negative for all ranks, with average values higher in younger nestlings (Figures 3C and 3D; Tables S19–S24). These results underscore the benefit of flexible biparental care for younger nestlings' growth and development.

## Discussion

Our high-resolution tracking of barn owl parents provides compelling evidence that biparental care in the wild is governed by a dynamic interplay of negotiation, cooperation, state-dependence, and temporal coordination. In support of H1 (partial compensation), we found that, when one parent (typically the male) delivered fewer prey or expended less foraging effort, the other (female) increased her own provisioning rate in response, but never fully offset the deficit. This nightly tit-for-tat aligns with game-theoretic models in which parents negotiate care levels in real time and predicts partial rather than complete compensation.^7^^,^^8^^,^^9^^,^^25^^,^^38^^,^^39^ At the same time, our results highlight remarkable flexibility in parental roles rather than strict role specialization. The female’s share of provisioning was highly variable across different nights and conditions (low repeatability), indicating that the chick provisioning investment of each parent is not rigidly set. As predicted by H2 (sex-specific role specialization), we observed that, although males generally provided more food under baseline conditions, females ramped up provisioning when brood demand was high or prey scarce. In fact, the division of labor shifted adaptively with context: females readily assumed greater foraging and provisioning duties when brood demand was high or when food was likely scarce around the nest, yet curtailed their efforts in prey-rich conditions (i.e., wildflower strips areas). When conditions were favorable, pairs tended toward a more asymmetric division of labor, with the male handling a larger share of provisioning and the female investing less. These context-dependent shifts from asymmetrical to more equitable sharing echoes predictions from specialization models and our H2: under low demand or abundant food, one parent can shoulder most tasks; under high demand, both must share labor to maximize brood success.^15^ This compensatory response by females is consistent with the hypothesis that biparental chick provisioning is more pronounced in challenging environments and when male provisioning is insufficient, supporting the adaptive value of flexible biparental roles in buffering environmental challenges.^5^^,^^22^ In this sense, we confirm the fundamental role of wildflower strips in deeply modified environments, such as agricultural and urban landscapes.^35^^,^^36^^,^^37^^,^^40^ Rather than a rigid division of labor, barn owl pairs employed a gradual, need-based redistribution of effort—essential for coping with fluctuating prey availability in nocturnal habitats.

Consistent with H3 (state-dependent investment), provisioning rates scaled positively with each parent’s energetic state: individuals in better condition or foraging in richer habitat reduced self-maintenance and increased care, whereas those in poorer condition conserved energy. When self-feeding rates rose or hunting habitat improved, parents—in particular females—scaled back provisioning, likely preserving energy for future reproductive opportunities.^17^^,^^20^ Conversely, low male hunting success, effort, high self-feeding and poor habitat triggered a compensatory uptick in female effort, reflecting the trade-off between self-maintenance and current brood care. Increased provisioning by females in biparental pairs incurs significant energetic costs, as reflected by elevated VeDBA values and decreased self-feeding rates. These findings suggest that, while biparental care enhances nestling growth, it also places substantial physiological demands on the more active parent.^41^^,^^42^ The correlation between VeDBA, a proxy for movement-related metabolic power,^43^ and reproductive success highlights the importance of balancing provisioning effort with self-maintenance, underscoring the physiological trade-offs inherent in biparental care.^44^ The observed reductions in self-feeding during intense provisioning periods may also confer indirect benefits, such as lower wing loading, which could reduce flight costs and potentially enhance foraging efficiency over time.^45^ Decisions to nest closer to suitable foraging grounds or closer to the peak of prey availability may mitigate these costs.^41^ Experimental work on burying beetles (*Nicrophorus vespilloides*) similarly showed that parents tailor their contributions according to both their own condition and their partner’s state.^46^ Such plasticity in response to personal and partner state reinforces the view that parental care decisions emerge from ongoing cost-benefit evaluations rather than fixed sex roles. Furthermore, by continuously monitoring nest visits and foraging departures, we show that parents’ feeding rates are mutually contingent not only via prey deliveries but also via the timing of their foraging trips. Males exhibit a steady decline in foraging probability as the night progresses, regardless of the female’s contribution. In contrast, female foraging schedules differ markedly with partnership balance: in pairs where females contribute little (low provisioning share), they abandon foraging sharply^34^—reducing trip-initiation probability by ∼75% within the first 2 h—whereas in more equitable pairs, females forage in parallel to males throughout the night. This pattern underscores that females in cooperative pairs incur additional time and energy costs, extending their hunting bouts to match their mate’s effort.

In line with H4 (coordinated foraging), direct coordination via short nest visits and increased encounter rate proved integral to provisioning dynamics. We showed that the parents’ feeding rates were influenced by each other’s behavior in real time possibly via their encounter rates at the nest. Frequent nest-relief events and in-nest interactions likely allowed parents to gauge each other’s recent success and adjust accordingly—akin to a quick status update that informs the next foraging decision. This pattern aligns with theoretical turn-taking models, in which conditional cooperation based on partner contributions emerges as a stable strategy.^24^ Such coordination could improve efficiency (avoiding both parents hunting at the same time unnecessarily) and may also have anti-predator benefits (e.g., synchronous nest visits might reduce overall activity around the nest^47^). While barn owls do not alternate with the precision seen in some songbirds,^24^^,^^48^ their asymmetric responsiveness—females reacting more strongly to male performance than vice versa—suggests that the parent with the most accurate information about brood status bears the greater responsibility for fine-scale negotiation.^9^ This asymmetry could stem from informational differences as the female, being the one who typically stays with the nestlings and feeds them directly (especially in the first two weeks of their life),^49^^,^^50^ might have better knowledge of the brood’s hunger level and thus respond more strongly to any deficit. In addition, parental effort (prey delivered per nestling) of one sex was positively related to the hunting effort of the other. These results would support that partners providing more food to their offspring have higher behavioral similarity, defined as the tendency for two individuals to behave like each other. A link between behavioral similarity and higher reproductive success has been experimentally demonstrated in monogamous animals including cichlid fish^51^ and birds.^52^^,^^53^^,^^54^

We also documented behavioral adjustments across successive nights confirming our H5 (sequential adjustment), predicting that past effort and resulting energy states influence current care strategies.^17^^,^^18^^,^^19^ Female provisioning share rose on nights following a previous low-share bout, reflecting a sequential adjustment in effort and provisioning also at the pair level. Such temporal coupling allows pairs to buffer against occasional shortfalls while avoiding chronic overexertion—a dynamic rhythm of “give and take” across nights that likely optimizes lifetime reproductive success in a variable environment.

Finally, in agreement with H6 (offspring fitness outcomes), pairs exhibiting a more equal provisioning ratio delivered more prey per nestling, boosting nestling survival and particularly ameliorating the disadvantage of later-hatched nestlings. In barn owls, as in many species with hatching asynchrony, the last-hatched nestlings often face a survival disadvantage due to size hierarchy and food competition.^55^^,^^56^^,^^57^ Our results suggest that increased biparental effort in nestling provisioning can mitigate this disadvantage—effectively buffering the younger nestlings from starvation by ensuring more equal food distribution even in a typically competitive brood hierarchy. One possible mechanism behind these observations is related to selective feeding by parents. For approximately the first 15 days after hatching, females remain in the nest, distributing the prey provided by the male to the nestlings.^49^ After this brooding period, females may choose to either join the male in chick provisioning, as shown in this study, or allow the male to provision alone.^34^ In the latter case, the male’s prey items may simply be left in the nest for the nestlings to consume independently.^58^ But when the female cooperates in provisioning, she may assist the younger nestlings in feeding facilitating the ingestion of bigger prey items.^59^^,^^60^^,^^61^ This interpretation does not preclude the possibility that highly efficient foraging males can adequately provision all nestlings on their own when resources near the nest are abundant.^62^ Intriguingly, we detected a slight negative association between male hunting success and nestling survival. This may reflect that nights of exceptional male hunting success coincided with reduced female provisioning, hinting at an indirect trade-off where solo male effort may come at the expense of broader coordination and nest attendance. Furthermore, because male prey deliveries per nestling are uncorrelated with his success rate, additional hunts may yield diminishing returns for chick provisioning, potentially explaining the marginal negative effect on survival. This highlights an important evolutionary benefit of retaining two active caregivers: brood outcomes are enhanced beyond what either parent could achieve alone. When both male and female fully engage in care, offspring gain not just increased food, but potentially a synergistic advantage (e.g., more consistent feeding, protection, and oversight).^15^ Theoretical and comparative studies have noted that species with high offspring demands often rely on biparental care, and that offspring can gain when both parents cooperate.^22^^,^^39^^,^^63^ This synergistic effect—where two active caregivers produce better outcomes than each alone—underscores the evolutionary value of maintaining dynamic, responsive biparental care.^2^^,^^16^^,^^22^ In the face of unpredictable nightly fluctuations in prey availability and environmental conditions, the ability of barn owl parents to negotiate, coordinate, and recalibrate their efforts in real time functions as a robust strategy to secure reproductive success.

Our study highlights the limitations of classic theoretical frameworks that model contributions to care as simple sex-specific traits and ignore behaviors taking place away from where offspring are fed. To further progress our understanding of parental care we need theory that incorporates newly measurable variables, such as hunting efficiency and effort, self-feeding, energy expenditure, encounter at and outside the nest, as well as time spent at the nest. Parental care is a suite of integrated, flexible behaviors that vary according to environmental and social factors, and we urge further theoretical and empirical studies to address the drivers of parental flexibility, particularly how foraging performance, partner behavior, parent-offspring interactions, and ecological conditions influence parental care strategies, survival, and fitness.^64^ Accurate, high-resolution behavioral data can help refine or reformulate existing theories,^28^ and will be crucial for testing new theories that are more ecologically explicit.^32^ A future comprehensive framework would perhaps bridge optimal foraging and parental negotiation models by incorporating both environmental and partner behavior decision functions.

In summary, our study demonstrates that offspring provisioning is a finely tuned collaboration shaped by conflict, cooperation, state-dependence, and temporal dynamics. We illustrate how biologging tools can reveal previously inaccessible details of parental care dynamics, illuminating the adaptive strategies employed by a socially monogamous species in response to varying environmental and social pressures. As biologging technology continues to advance, our approach offers an empirical example for studying flexible parental care in chick provisioning across species, contributing to a broader understanding of how ecological and social factors shape reproductive success in changing environments.

### Limitations of the study

While our simultaneous GPS-accelerometer biologging approach offers valuable insight into fine-scale parental provisioning dynamics, several caveats merit consideration. First, despite having tested that there was no difference in average reproductive outcome and weight between tagged and non-tagged birds, logger deployment may always alter natural behavior in ways that researchers cannot quantify. Second, our sample is limited to a short period of time, which can only give us a partial understanding of the parental behavior over the nestling rearing time. Third, we did not focus on environmental variables other than wildflower strips area as proxy of optimal hunting grounds and resource availability; hence, there might have been other external variables (e.g., temperature, precipitation, moon illumination) that could affect the instantaneous behavior. Finally, our focus on a single population in a specific landscape limits generality: behavioral rules uncovered here may vary across different barn owl populations or other species with distinct ecological contexts. Future work combining biologging with direct nest observations, experimental manipulations of parental load, and multi-population comparisons will help address these limitations and refine our understanding of biparental care flexibility under natural conditions.

## Resource availability

### Lead contact

Inquiries regarding research site, methods, data, code, results, and any other matter related to the manuscript should be addressed to the corresponding author, Paolo Becciu (paolobecciu@protonmail.com), who will gladly answer them.

### Materials availability

This is an observational, biologging-based study, which did not use any materials or agents and did not generate new ones.

### Data and code availability

• Raw GPS-accelerometer tracking datasets are deposited in Movebank (www.movebank.org), under the project named “Barn owl (Tyto alba),” ID 231741797, at https://www.movebank.org/cms/webapp?gwt_fragment=page=studies,path=study231741797.
• User-friendly processed tables are deposited in GitHub available at https://github.com/paolobecciu/flexbiparentality-barn-owls.
• All original R scripts are deposited in GitHub and available at https://github.com/paolobecciu/flexbiparentality-barn-owls.

## Acknowledgments

The authors would like to thank R. Séchaud, L. Ancay, A.P. Machado, R. Bühler, C. Gémard, C. Massa, A. C. Heinz, S. Zurkinden, N. Külling, M. Froehli, and all the field assistants, students, and interns from University of Lausanne and the Swiss Ornithological Institute for their help in collecting field data. The authors are grateful to R. Allemann for her barn owl drawings used in the graphical abstract. This study was financially supported by the 10.13039/501100001711Swiss National Science Foundation (grants no. 310030_200321). The authors also want to thank all the members of the Barn Owl Research Group for feedback on this study. Finally, we thank M. Kavelaars and an anonymous reviewer for their insightful comments on an earlier version of this manuscript.

## Author contributions

P.B.: Conceptualization, data curation, formal analysis, investigation, methodology, visualization, writing – original draft preparation, writing – review & editing.

K.S.: Data curation, formal analysis, investigation, methodology.

E.M.: Formal analysis, investigation, writing – review & editing.

J.L.S.: Validation, writing – review & editing.

A. Romano.: Validation, writing – review & editing.

B.A.: Validation, funding acquisition, resources, writing – review & editing, supervision.

A. Roulin.: Funding acquisition, resources, writing – review & editing, supervision.

## Declaration of interests

The authors declare no competing interests.

## Declaration of generative AI and AI-assisted technologies in the writing process

During the preparation of this work the lead author used ChatGPT in order to improve readability. After using this tool, the authors reviewed and edited the content, and take full responsibility for the content of the publication.

## STAR★methods

### Key resources table

**Table:** 

REAGENT or RESOURCE	SOURCE	IDENTIFIER
**Deposited data**		

Tracking data	https://www.movebank.org/cms/webapp?gwt_fragment=page=studies,path=study231741797	–

**Software and algorithms**		

Code and processed data	https://github.com/paolobecciu/flexbiparentality-barn-owls	–

### Experimental model and subject details

Barn owls are a medium sized bird. Adults used in our study have a mean ± SD of 322 ± 22.6 g (females) and 281 ± 16.5 g (males). Adult sex (based on the presence or absence of a brood patch) and age (yearlings or older, based on plumage) were recorded at the first visit and confirmed at the second visit. Barn owls were monitored from egg laying to fledging to gather data on their reproduction success. Number of eggs, nestlings, and body measurements were taken as part of annual monitoring of the species.^50^^,^^65^

Permits for capturing, handling, and attaching animal tracking devices to barn owls in Switzerland were issued by the authorities of the Department of the Consumer and Veterinary Affairs (legal authorizations: VD, FR and BE 3213 and 3571; capture and ringing permissions from the Federal Office for the Environment).

### Method details

#### Study area, data collection and tag deployment

The study was conducted in the Western Swiss plateau, an area of 1,000 km^2^ characterized by an open and largely intensive agricultural landscape, where a wild population of barn owls breeds in nest boxes.^65^^,^^66^ Between March and August in 2019 and 2020, 163 breeding barn owls (84 females and 79 males) were equipped with AXY-Trek Mini loggers (Technosmart, Italy). We deployed 92 loggers in 2019 and 71 in 2020 (see Schalcher et al.^67^). Approximatively 25 days after the first egg hatched, parent barn owls were captured using automatic sliding traps at their nest sites. During the initial capture for tag attachment (∼30 min) birds were also ringed, weighed, and standard morphometric measurements (wing length, tarsus length) were taken. At tag recovery, handling was considerably shorter (3–5 min) and only involved tag removal and weighing. The averaged body mass from both visits was used for subsequent analysis of body condition (see below). Loggers were attached as backpacks using a Spectra tube harness (Bally Ribbon Mills, USA), and recorded GPS data (1 Hz) from 30 min before sunset until 30 min after sunrise, and accelerometer data (50 Hz) continuously (triaxial recording range ±16 g, 10-bit resolution). After 10 ± 2 days, loggers were recovered by recapturing the owls. The bio-loggers recorded data for 5 nights on average (±1 night). Each device weighed 12.4 ± 0.1 g, corresponding to 4% on average of the barn owl’s total body mass (min = 3%, max = 5%), which we considered reasonable given the short deployment period^67^ (see below). For this study we selected breeding partners with overlapping tracking periods (136 individuals in 68 pairs). Among males, eight individuals were tagged in both years; among females, five were tagged in both years, with an additional two females tagged twice within 2019 (during their first and second broods). Only two pairs maintained the same male-female combination and were tagged in both years. In addition, motion-sensitive camera traps (Reconyx HC500 hyperfire) positioned at the entrance of all nest boxes documented prey deliveries to the nest.^68^ These cameras were configured to record bursts of three images upon detecting motion, allowing us to reliably capture evidence of prey deliveries. These data were used to confirm prey deliveries classified using the GPS and accelerometer data, as detailed in Schalcher et al.^67^

We calculated a body condition variable for each parent using the Scaled Mass Index (SMI),^69^^,^^70^ which better accounts for allometric scaling between body mass and structural size compared to simple ratios or residuals. We calculated SMI from the weight and wing length measured at the moment of tagging the parents. To assess potential deleterious effects of GPS deployment, we also compared the Scaled Mass Index (SMI) of individuals at attachment and recovery of data loggers using a linear mixed model (LMM) for each sex. Our analysis revealed a statistically significant negative trend in SMI for females (estimate = −11.954, *p* = 0.042, *n* = 134) but not for males (estimate = −2.262, *p* = 0.523, *n* = 134). Given that females are significantly larger than males (LMM: estimate = −47.311, *p* < 0.001), if the tags were imposing a harmful effect (e.g., through additional weight), we would expect a more pronounced negative trend in males. Therefore, this pattern indicates that the observed decline in female SMI is unlikely to be attributed to the tag to body mass ratio, but likely to her gradual weight loss due by resuming hunting after the incubation and brooding at the nest.^50^

### Quantification and statistical analysis

Data handling, calculation of parameters, variables and statistical analyses were performed in the statistical environment R 4.3.2^68^ with RStudio as graphic user interface.^71^ Underlying code is available at https://github.com/paolobecciu/flexbiparentality-barn-owls.

#### Behavioral classification and variables

We classified barn owl behaviors (flight, landing, hunting strikes, self-feeding) using acceleration and GPS data.^67^ Behaviors were summarized at 1-s intervals and linked to the nearest GPS location in time. Behavioral classifications used the raw acceleration data, the Vectorial Dynamic Body Acceleration (VeDBA – a summary metric of body motion^43^), and body pitch angle (see details in Schalcher et al.^67^). Successful hunts were confirmed by nest box camera data and GPS records showing direct flights back to the nest after a hunting attempt or a self-feeding event. Unsuccessful strikes were inferred from hunting strikes followed by one or more other hunting strikes.^67^

Biparental care in chick provisioning was calculated as the proportion of prey brought by the female relative to the total prey brought by both parents per night (nightly female provisioning share) and over the tracking period (mean female provisioning share). A proportion of 0.5 indicates equal contribution, 0 indicates no contribution from the female (only paternal provisioning), and 1 indicates all prey were provided by the female. Male’s provisioning share is given by 1 – p (with p representing the female provisioning share). We focused on the female’s contribution owing to the greater variability in female provisioning behavior.^34^^,^^50^

We assessed several parameters at night and individual scale to understand parental provisioning contributions.

(1) Prey per nestling: number of preys delivered per night divided by brood size at tag recovery by male (prey.per.chickM), female (prey.per.chickF) and both parents (prey.per.chick).
(2) Energy expenditure and hunting effort: measured by total hunting attempts (hunt.att.sumM and hunt.att.sumF), and average Vectorial Dynamic Body Acceleration or simply VeDBA (avg.vedbaM and avg.vedbaF).
(3) Hunting success rates: measured dividing successful hunting attempts by total attempts (hunt.succ.rateM and hunt.succ.rateF) to assess hunting efficiency.
(4) Prey eaten (refuelling): we also recorded the number of preys eaten per night and used it to calculate the proportion of prey eaten out of the total captured (prop.eaten.capturedF and prop.eaten.capturedM) to assess refuelling behavior.

Furthermore, we calculated the distance between parents at every timestamp and defined an encounter as any instance where parents were less than 20 m apart (i.e., within the GPS error margin). To avoid over-counting brief departures and returns, successive encounters were counted when separated by a 300-second threshold. We then categorized these encounters into two groups: those occurring at the nest (i.e., when both parents were within 20 m of the nest) and those outside the nest. In addition, we computed the total time each parent spent at the nest per night. We also used a lagged value (to the previous night) of all behavioral variables regarding parental provisioning and hunting effort to test the night-to-night sequential adjustments.

#### Environmental variables

To test if female provisioning share changed with different habitat features, we calculated the area and distance to several landscape features within a 1.5 km radius around the nest box, encompassing the local barn owl population’s average home range (7 km^2^).^35^^,^^66^^,^^72^ We extracted 11 habitat features, as the main landscape features in the study area: distance to road, distance to forest, distance to urban settlement, urban area, meadow area, hedgerow area, wildflower strips area, extensively used pasture area, extensive crop area, urban density (urban area divided by total building area), and crop diversity (number of different crops divided by total crops). Areas are expressed in km^2^ and distances in meters. We focused on area of wildflower strips, which are considered to promote biodiversity^37^^,^^40^ and positively selected by foraging barn owls during breeding^35^ and wintering period.^36^ We confirmed the importance of wildflower strips as a key environmental feature by running univariate Bayesian Generalised Linear Models (GLMMs) estimated using MCMC sampling (3 chains of 20,000 iterations, 5,000 warmups; “brms” package in R^73^) for each environmental feature to test which best predicted female provisioning share (Figure S7; Table S3). Model comparison used leave-one-out cross-validation (function ‘loo’ from package “loo” in R^74^) returning the difference of expected log predictive density (ELPD_diff) between the model with higher expected log predictive density (elpd) and the other models elpds.^74^ We then included the environmental variable from the best univariate model in multivariate models to test our predictions (see “statistical analysis” section).

Agricultural landscape features were provided by the “Direction Générale de l’Agriculture, de la Viticulture et des Affaires Vétérinaires” of the states of Vaud and Fribourg, and urban features were retrieved using the TLM3D catalog of the Swiss Federal Office (Swiss Topographic Landscape Model - https://www.swisstopo.admin.ch/en/landscape-model-swisstlm3d). Landscape features were calculated using the R package ‘sf’.^75^

We used year as an environmental factor in our analyses to control for annual variations in resource availability. Barn owl breeding performance is closely tied to prey abundance, particularly common voles (*Microtus arvalis*), which fluctuate annually.^76^^,^^77^ In our study area, 2019 was worse than 2020 in terms of clutches recorded (*n* = 62 and *n* = 104, respectively) and average prey (genus *Microtus*) found in nests (3.13 in 2019, 4.77 in 2020).

#### Statistical analysis

We tested repeatability in female provisioning share within pairs and across nights running a mixed effect model using data at night scale, with the PairID as random effect, year and brood.size as fixed effect, using the package “rptR” with the function ‘rpt’.^78^ This allowed us to estimate the adjusted repeatability, and its confidence intervals estimated by parametric bootstrapping (n_boost_ = 1000).^78^ We tested correlation among parental provisioning variables (female provisioning share, prey per nestling by both parents, by male only and by female only) using the package “BayesFactor” with the function ‘correlationBF’ (iterations = 10,000). To test if female provisioning share was related to parent hunting behavior, energy expenditure and environmental conditions, we used Bayesian GLMMs estimated using MCMC sampling (3 chains of 20,000 iterations, 5,000 warmup). The nightly female provisioning share was predicted as a function of male and female hunting behaviors (sum of hunting attempts, hunting success proportion, self-feeding proportion, mean VeDBA), time spent at the nest by each parent, encounters between parents at and out of the nest, body condition (SMI) of each parent at tagging, area of favorite hunting ground (area_wildflower.zsqrt), year, nestling loss during the tagging period (brood.size.change01F) and age of youngest nestling at tagging (as proxy of nestling rearing stage and brood size). Pair ID was used as a random intercept. All continuous explanatory variables were standardized (z-scores). Bayesian modeling was performed using “brms” package in R.^73^^,^^79^ Posterior means were used as estimates, with 2.5% and 97.5% quantiles as the upper and lower bounds of the 95% credible interval (CrI). Effects were considered meaningful when the 95% CrI did not contain 0. Similarly, we analyzed female provisioning per chick (prey.per.chickF) as a function of male hunting behaviors, area of favorite hunting ground, and year. The same model was used for prey.per.chickM as a function of female hunting behaviors. Also, we modeled the prey per nestling provided by male and female combined (prey.per.chickMF) as a function of the female provisioning share and parents’ hunting behaviors. The combination of these four models would reveal key relationships between parent investment relative to their social (partner) and physical environment (area of favorite hunting ground).

Furthermore, we evaluated the probability of foraging at each timepoint of the night by both male and female partners until ending of the nightly foraging activity (binomial variable associated with time: 1 as foraging trip and 0 as the termination of foraging activity each night), in relation to their chick provisioning (female provisioning share). This highlighted foraging patterns from both parents over the course of the night, and we expected to see variation in both sexes according to the effort of their partner (i.e., prolonging or stopping foraging). To achieve this, we built a time-to-event Piece-wise Exponential Additive Mixed Model (PAMM) for each sex separately using the “pammtools” package in R.^80^ This method combines the flexibility of Generalised additive Mixed Models (GAMM) with Piece-wise exponential Models (PEM) as an alternative to Cox models used in survival analysis. Through this we estimated the probability of foraging at any given timepoint during the night and compared this probability within sexes by their female provisioning share, as well as visually between sexes.

To test if mean female provisioning share would affect nestling growth, especially in larger broods with greater size difference between the oldest and the youngest nestling, we ran Bayesian GLMMs (as above). These models assessed the nestling’s body weight and wing length as functions of the time of measurement (3-level factor: before tagging, after tagging, before fledging), nestling rank (4-level factor: 1 = born first, 2 = second, 3 = third and 4 = born fourth or younger), mean female provisioning share and the three-way interaction of these terms. By evaluating the interaction, we observed differences in nestling sizes across ranks and at different times across the female provisioning gradient. Nestling weight and wing length change were also modeled in the same way with the difference that the time of measurement included only two groups since the response variables reflected a change between two periods: a value for the change *before tagging* and *after tagging*, and another for the change between *after tagging* and *before fledging*). We used the function ‘emtrends’ (estimated marginal means of linear trends) to retrieve the estimated slopes in our three-way interaction and ‘pairs’ to estimate pairwise comparisons between groups in the R package “emmeans”.^81^ The summary for estimated marginal means shows the median of the posterior distribution of each estimate (MPD), along with highest posterior density (HPD) intervals.

In addition, to test effects on nestling survival (nestlings counted at tag recovery divided by number of eggs) we constructed a series of Bayesian GLMs for each group of factors—clustering factors related to mean female provisioning share—including year in all models. We built a model to account for combined parental care using mean female provisioning share, their combined provisioning per nestling, and the area of wildflower strips (preferred hunting ground) around their nest.

From each model we reported MPD (median of the posterior distribution), 95% CrI (95% credible interval) extracted with the “bayestestR” package in R.^82^ Bayesian model checking of predictive ability was done visually using the ‘pp_check’ function (500 simulated draws) in “bayesplot” package in R.^83^
