# Supplementary material for: Real-time context-dependent cooperation in parental provisioning reveals fitness payoffs in barn owls
Source: iScience. 2025 Sep 10;28(10):113533. doi: 10.1016/j.isci.2025.113533 (PMC12489757; doi:10.1016/j.isci.2025.113533)
Supplement: Document S1. Figures S2–S12 and Tables S1–S24 [file mmc1.pdf]

iScience, Volume *28*

Supplemental information

Real-time context-dependent cooperation in parental provisioning reveals fitness payoffs in barn owls

Paolo Becciu, Kim Schalcher, Estelle Milliet, James L. Savage, Andrea Romano, Bettina Almasi, and Alexandre Roulin

Supplementary materials

	Male	Female	Pair
**Prey per nestling**	1.86	0.67	2.53
**Provisioning share***	0.73	0.27	
**Sum of hunting attempts**	41.26	20.41	
**Proportion hunting success**	0.31	0.27	
**Self-feeding****	0.18	0.21	
**Encounters - all**			5.470
**Encounters - nest**			4.283
**Encounters - out**			1.187
**Time at the nest (mins)**	68.789	95.657	
**Body condition (SMI)**	281.580	329.261	

*This value is the female provisioning share (main text), which is a pair value shown from the male and female share perspective. **Proportion of prey eaten on total prey captured.

Table S1. Overall nightly mean by sex and by pair of all individuals combined (n_{ID} = 136).

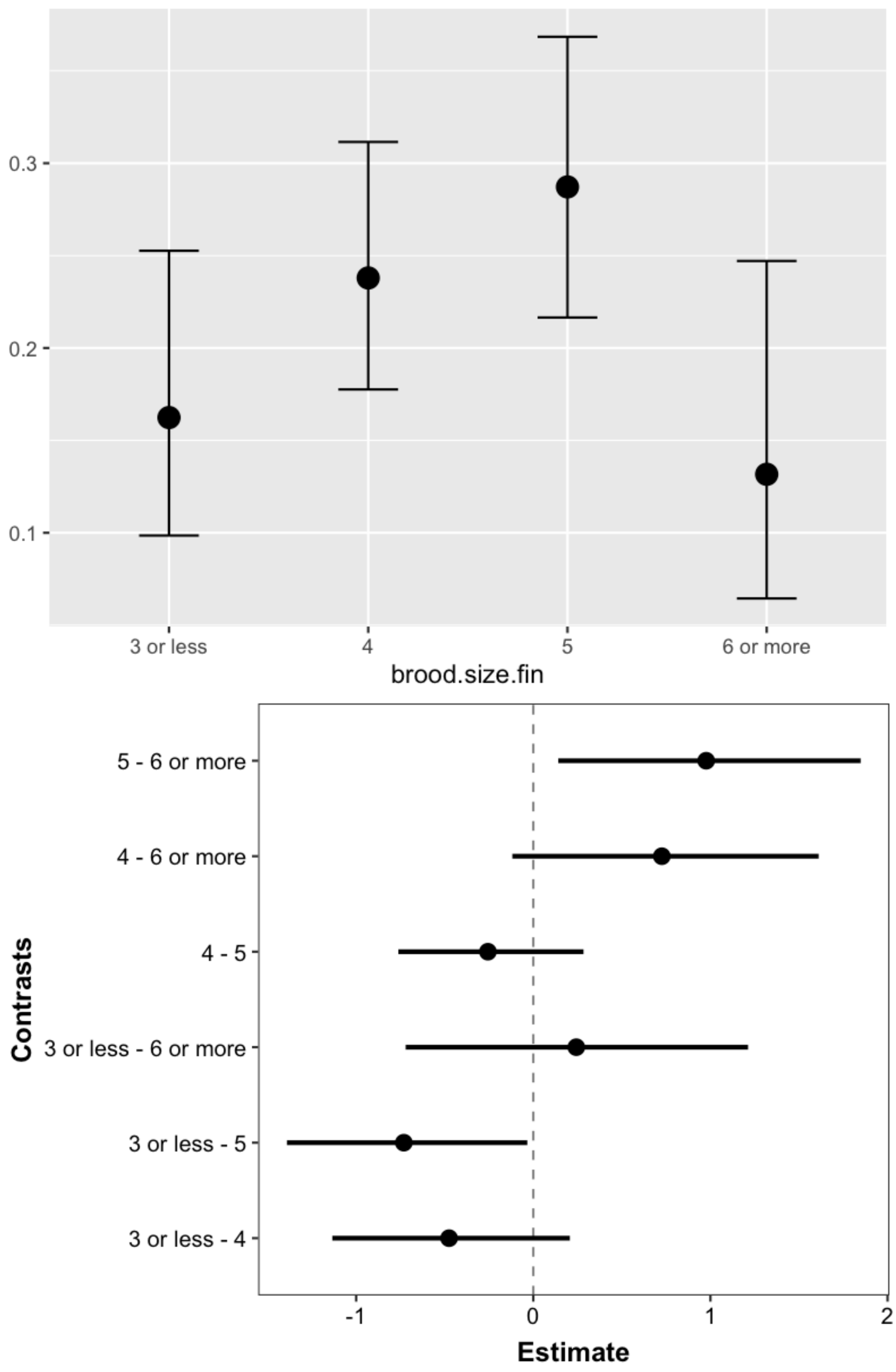

Figure S2. Top: Female provisioning share changing across brood sizes (posterior mean and 95% Cr.I. of the univariate Bayesian GLMM). Bottom: pairwise comparisons between brood size groups. Sample size per brood size group: $n_{\text{“3 or less”}}$ = 11; $n_{\text{“4”}}$ = 26; $n_{\text{“5”}}$ = 25; $n_{\text{“6 or more”}}$ = 6.

brood.size_ pairwise	estimate	lower.HPD	upper.HPD
3 or less – 4	-0.475	-1.134	0.2066
3 or less – 5	-0.731	-1.391	-0.0336
3 or less - 6 or more	0.243	-0.721	1.2126
4 – 5	-0.256	-0.762	0.2836
4 - 6 or more	0.726	-0.118	1.6111
5 - 6 or more	0.976	0.141	1.8483
Point estimate displayed: median			
Results are given on the log odds ratio (not the response) scale.			
HPD interval probability: 0.95			

Table S2. Pairwise comparisons of female provisioning share differences between brood sizes (see Figure S2).

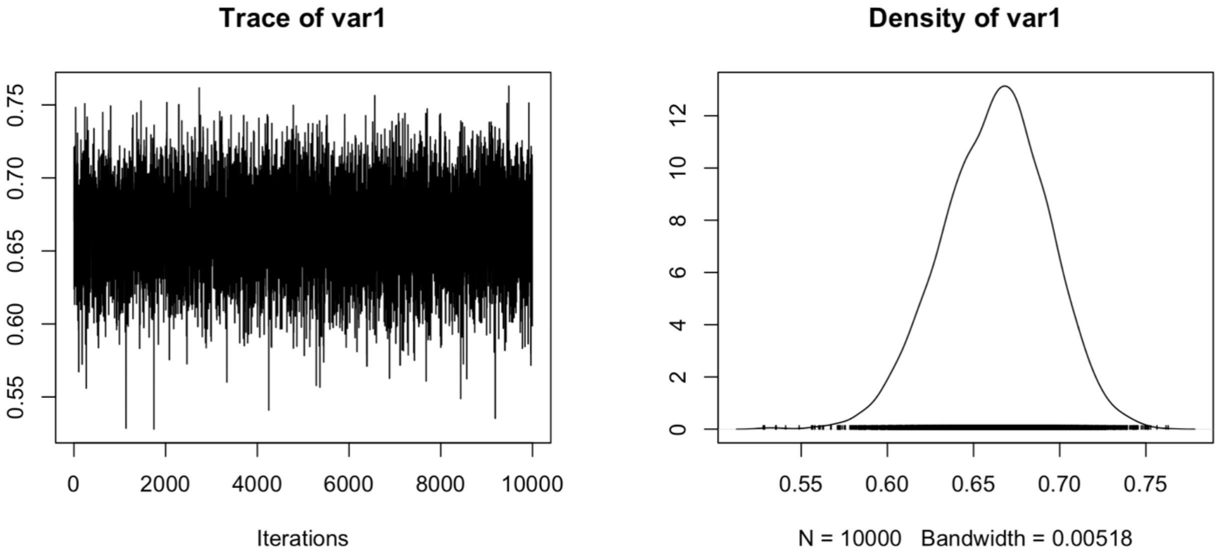

Figure S3. Posterior distribution of correlation coefficients between female provisioning share and prey per nestling brought by the female partner at nightly scale (rho = 0.66, 95%Cr.I. = [0.60, 0.72], iterations = 10,000).

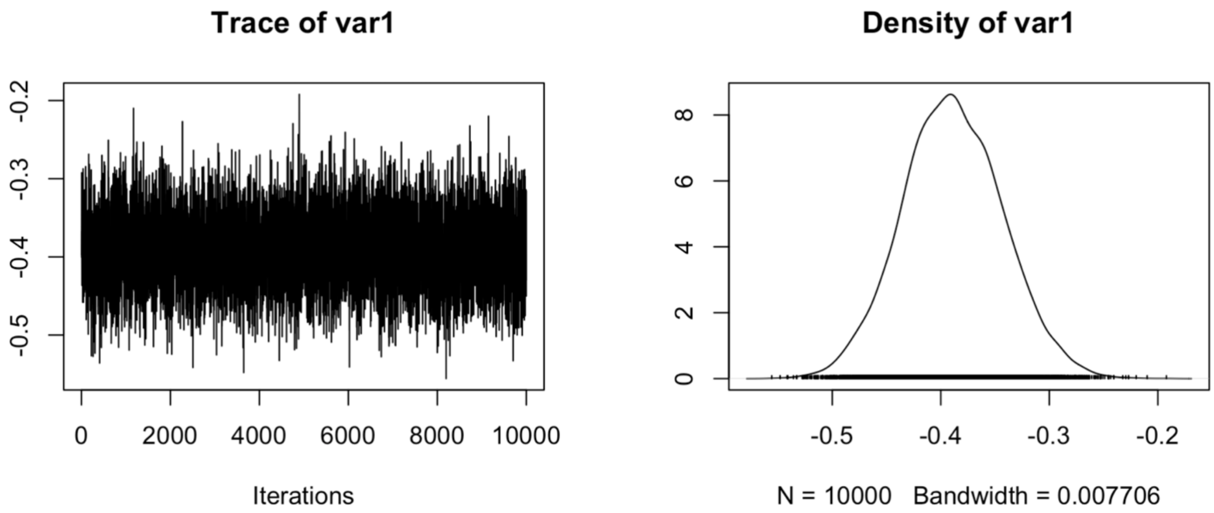

Figure S4. Posterior distribution of correlation coefficients between female provisioning share and prey per nestling brought by the male partner at nightly scale (rho = -0.39, 95%Cr.I. = [-0.47, -0.29], iterations = 10,000).

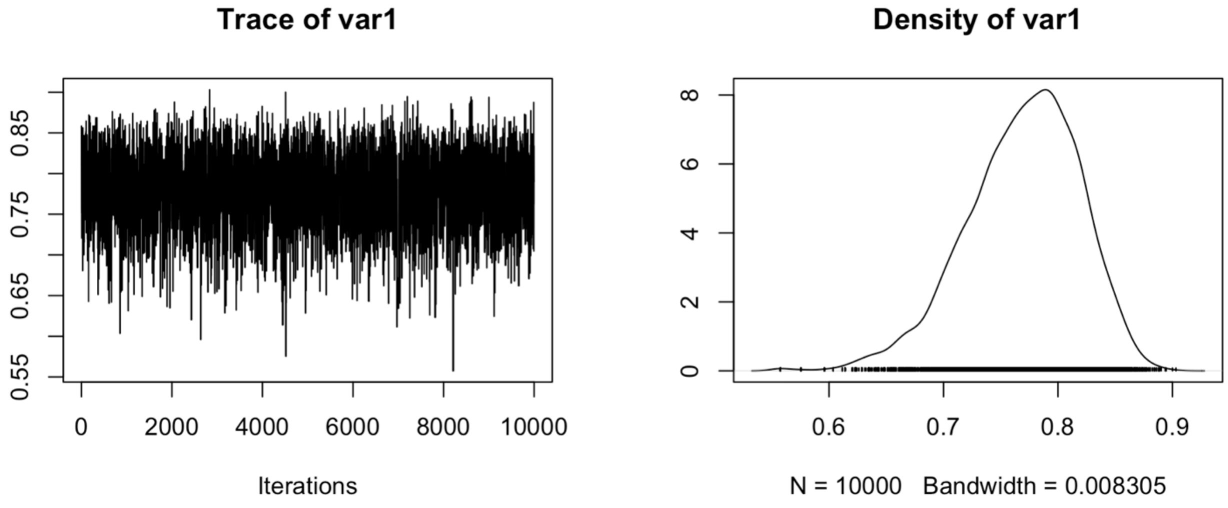

Figure S5. Posterior distribution of correlation coefficients between female provisioning share and prey per nestling brought by the female partner at individual scale (rho = 0.77, 95%Cr.I. = [0.66, 0.85, iterations = 10,000).

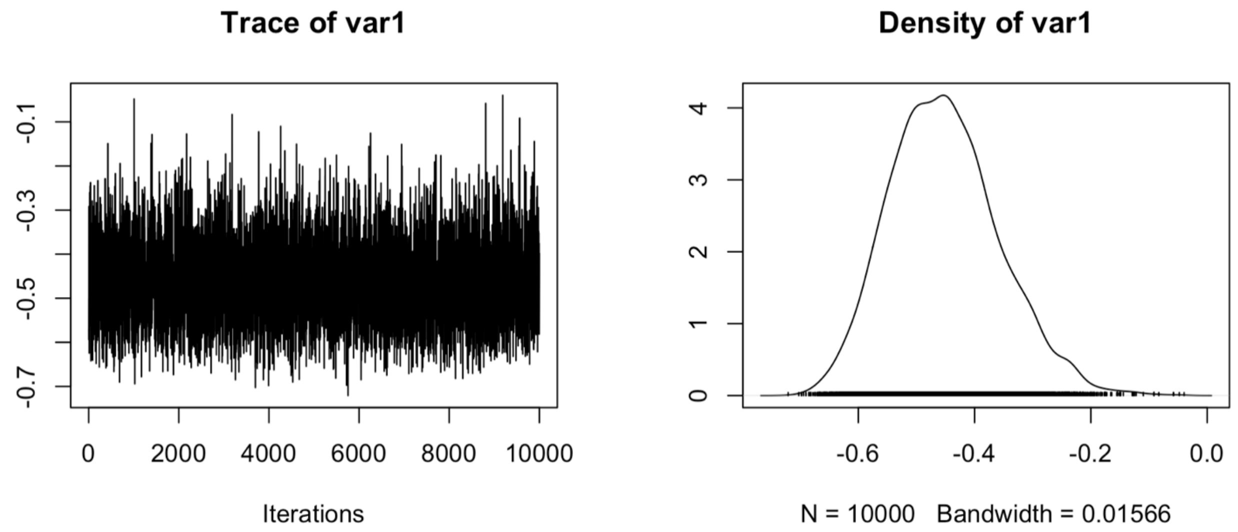

Figure S6. Posterior distribution of correlation coefficients between female provisioning share and prey per nestling brought by the male partner at individual scale (rho = -0.45, 95%Cr.I. = [-0.62, -0.25, iterations = 10,000).

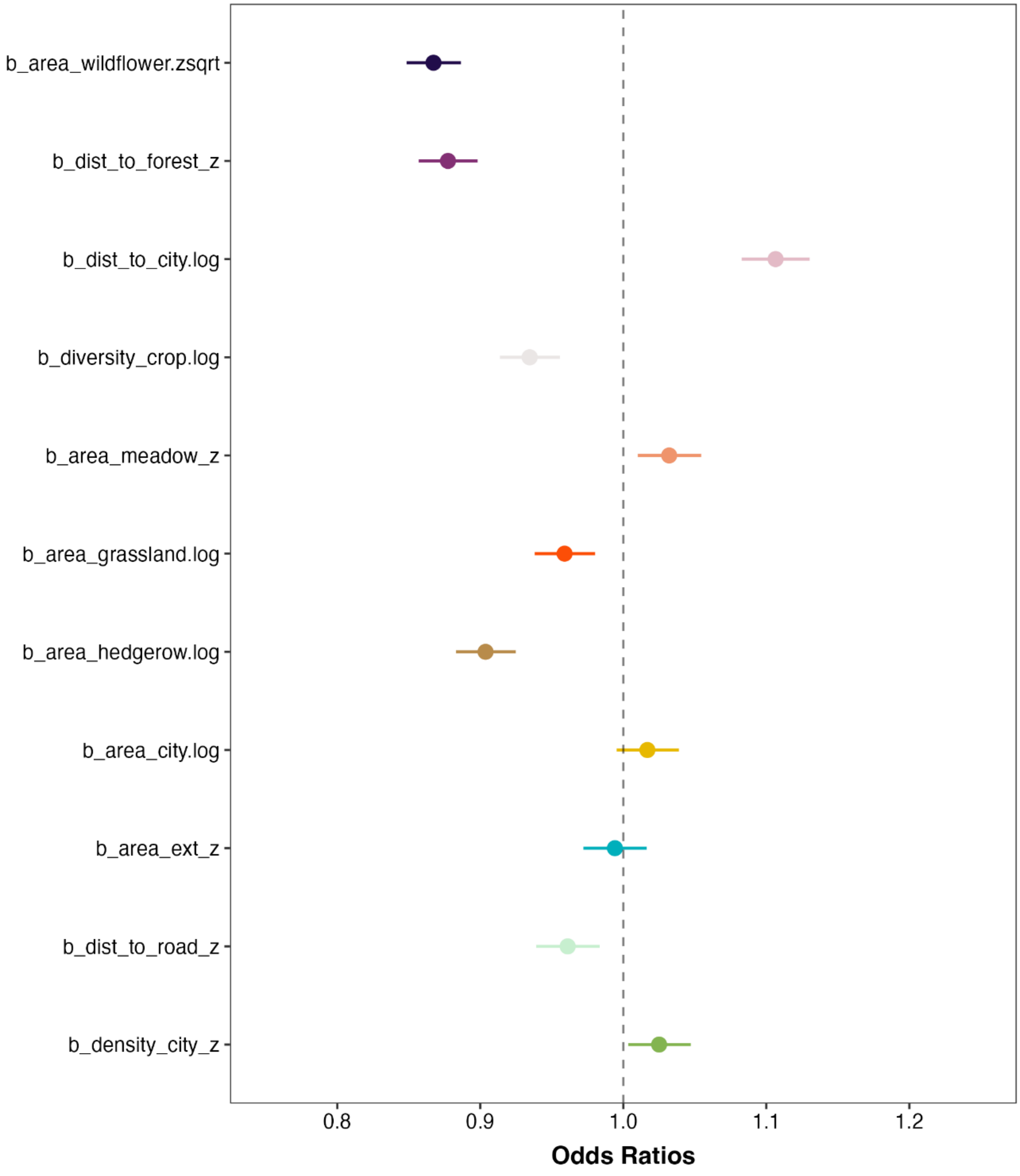

Figure S7. Univariate model coefficients of habitat feature predictors related to female provisioning share. Models are ordered by the best predictive to the worst, reflecting the model selection reported in Table S1 below. All variables including "_z" or ".z" are continuous variables standardised as z-scores. If they include "log" or "sqrt" mean that they have been log- or sqrt-transformed. These variables are listed and explained in the STAR*Methods sub-section "Environmental variables" of the main text.

Model comparisons:			
	elpd_diff	se_diff	variable
modelIDenv1	0.0	0.0	wildflower area
modelIDenv3	-26.0	141.1	distance to forest
modelIDenv8	-33.8	129.4	distance to city/settlement
modelIDenv11	-63.7	109.7	crop diversity
modelIDenv10	-66.0	107.8	meadow area
modelIDenv6	-66.0	97.0	grassland area
modelIDenv7	-70.6	90.5	hedgerow area
modelIDenv5	-74.5	101.9	city/settlement area
modelIDenv9	-78.5	96.2	extensive pasture area
modelIDenv2	-78.7	94.8	distance to road
modelIDenv4	-84.5	107.1	distance to city/settlement

Table S3. Model comparison between univariate models predicting relationship between pair female provisioning share and habitat features (see Figure S7), to justify the use of wildflower strips area in our Bayesian GLMM reported in our models in the main texts.

Parameter	Median	Mean	MAP	95% CrI
(Intercept)	-1.11	-1.11	-1.10	[-1.30, -0.91]
vedba.avg.F_z	0.10	0.10	0.10	[0.06, 0.14]
hunt.att.sum.F_z	0.28	0.28	0.28	[0.23, 0.33]
prop.refuelF_z	-0.25	-0.25	-0.25	[-0.30, -0.21]
prop.succ.dives.F_z	0.24	0.24	0.24	[0.20, 0.29]
vedba.avg.M_z	-0.06	-0.06	-0.05	[-0.11, -0.01]
hunt.att.sum.M_z	-0.24	-0.24	-0.25	[-0.29, -0.20]
prop.succ.dives.M_z	-0.27	-0.27	-0.26	[-0.31, -0.22]
prop.refuelM_z	0.08	0.08	0.08	[0.05, 0.12]
n_nest_encounters_z	0.14	0.14	0.14	[0.08, 0.20]
n_out_encounters_z	0.02	0.02	0.02	[-0.02, 0.05]
time_at_nest_M_z	-0.05	-0.05	-0.06	[-0.10, -0.01]
time_at_nest_F_z	-0.12	-0.12	-0.12	[-0.19, -0.05]
SMI_M_z	-0.04	-0.04	-0.04	[-0.17, 0.09]
SMI_F_z	-0.13	-0.13	-0.13	[-0.27, 0.00]
area_wildflower.zsqrt	-0.13	-0.13	-0.13	[-0.26, 0.00]
yearF2020	-4.78e-03	-4.99e-03	-0.01	[-0.28, 0.26]
brood.size.change01F1	-0.05	-0.05	-0.04	[-0.30, 0.20]
youngest.chick.age_z	-0.01	-0.01	-9.88e-04	[-0.19, 0.17]
Random effects SD/Cor: BroodID	0.48	0.48	0.48	[0.39, 0.61]

Table S4. Female provisioning share as response variable (N = 300). All parameters including "_z" or ".z" are continuous variables standardised as z-scores. Parameters including "M" or "F" refer to males and females, respectively, if the letter identifying the sex is missing it means that is relative to both parents combined. Parameters are listed and explained in the STAR*Methods.

Parameter	Median	Mean	MAP	95% CrI
(Intercept)	2.83	2.84	2.83	[2.65, 3.02]
prop.bip_z	0.12	0.12	0.12	[0.00, 0.24]
vedba.avg.F_z	0.08	0.08	0.08	[-0.02, 0.17]
hunt.att.sum.F_z	0.24	0.24	0.24	[0.13, 0.36]
prop.refuelF_z	-0.06	-0.06	-0.06	[-0.14, 0.03]
prop.succ.dives.F_z	-3.89e-03	-3.72e-03	-4.59e-03	[-0.09, 0.09]
vedba.avg.M_z	0.14	0.14	0.15	[0.05, 0.24]
hunt.att.sum.M_z	0.68	0.68	0.67	[0.57, 0.78]
prop.succ.dives.M_z	0.38	0.38	0.38	[0.28, 0.47]
prop.refuelM_z	-0.09	-0.09	-0.09	[-0.16, -0.01]
n_nest_encounters_z	0.30	0.30	0.30	[0.16, 0.43]
n_out_encounters_z	0.08	0.08	0.08	[-0.01, 0.17]
time_at_nest_M_z	0.07	0.07	0.07	[-0.03, 0.17]
time_at_nest_F_z	-0.08	-0.08	-0.08	[-0.22, 0.06]
SMI_M_z	0.06	0.06	0.07	[-0.06, 0.18]
SMI_F_z	0.10	0.10	0.11	[-0.02, 0.22]
area_wildflower.zsqrt	0.08	0.08	0.08	[-0.04, 0.20]
yearF2020	-0.22	-0.22	-0.22	[-0.47, 0.03]
brood.size.change01F1	-0.45	-0.45	-0.45	[-0.69, -0.22]
youngest.chick.age_z	0.23	0.23	0.23	[0.06, 0.40]
Random effects SD/Cor: BroodID	0.37	0.38	0.37	[0.28, 0.49]

Table S5. Prey per nestling delivered by both parents as response variable (N=300). All parameters including "_z" or ".z" are continuous variables standardised as z-scores. Parameters including "M" or "F" refer to males and females, respectively, if the letter identifying the sex is missing it means that is relative to both parents combined. Parameters are listed and explained in the STAR*Methods.

Parameter	Median	Mean	MAP	95% CrI
(Intercept)	0.78	0.78	0.78	[0.63, 0.92]
vedba.avg.M_z	0.04	0.04	0.04	[-0.03, 0.10]
hunt.att.sum.M_z	0.13	0.13	0.13	[0.06, 0.19]
prop.succ.dives.M_z	3.32e-03	3.28e-03	4.36e-03	[-0.06, 0.06]
prop.refuelM_z	0.02	0.02	0.02	[-0.03, 0.07]
time_at_nest_M_z	0.05	0.05	0.05	[-0.01, 0.12]
SMI_M_z	0.02	0.02	0.02	[-0.08, 0.12]
n_nest_encounters_z	0.06	0.06	0.06	[-0.01, 0.13]
n_out_encounters_z	0.03	0.03	0.03	[-0.02, 0.09]
area_wildflower.zsqrt	-0.09	-0.09	-0.08	[-0.18, 0.01]
yearF2020	-0.04	-0.04	-0.03	[-0.24, 0.16]
brood.size.change01F1	-0.20	-0.20	-0.19	[-0.39, -0.01]
youngest.chick.age_z	0.07	0.07	0.07	[-0.06, 0.21]
Random effects SD/Cor: BroodID	0.33	0.34	0.33	[0.26, 0.43]

Table S6. Female prey per nestling as response variable (N=325). All parameters including "_z" or ".z" are continuous variables standardised as z-scores. Parameters including "M" or "F" refer to males and females, respectively, if the letter identifying the sex is missing it means that is relative to both parents combined. Parameters are listed and explained in the STAR*Methods.

Parameter	Median	Mean	MAP	95% CrI
(Intercept)	2.00	2.00	2.00	[1.78, 2.21]
vedba.avg.F_z	-0.03	-0.03	-0.03	[-0.14, 0.08]
hunt.att.sum.F_z	0.28	0.28	0.28	[0.16, 0.40]
prop.succ.dives.F_z	-0.08	-0.08	-0.08	[-0.18, 0.01]
prop.refuelF_z	0.04	0.04	0.04	[-0.06, 0.14]
time_at_nest_F_z	-0.11	-0.11	-0.10	[-0.28, 0.06]
SMI_F_z	0.11	0.11	0.11	[-0.04, 0.25]
n_nest_encounters_z	0.40	0.40	0.40	[0.25, 0.55]
n_out_encounters_z	0.12	0.12	0.13	[0.02, 0.22]
area_wildflower.zsqrt	0.20	0.20	0.20	[0.06, 0.34]
yearF2020	-0.06	-0.06	-0.04	[-0.36, 0.24]
brood.size.change01F1	-0.25	-0.25	-0.25	[-0.52, 0.03]
youngest.chick.age_z	0.16	0.16	0.16	[-0.04, 0.37]
Random effects SD/Cor: BroodID	0.45	0.45	0.45	[0.32, 0.59]

Table S7. Male prey per nestling as response variable (N=302). All parameters including “_z” or “.z” are continuous variables standardised as z-scores. Parameters including “M” or “F” refer to males and females, respectively, if the letter identifying the sex is missing it means that is relative to both parents combined. Parameters are listed and explained in the STAR*Methods.

Parameter	Median	Mean	MAP	95% CrI
(Intercept)	-0.95	-0.95	-0.95	[-1.14, -0.75]
prop.bip_z_prev	-0.13	-0.13	-0.13	[-0.19, -0.07]
vedba.avg.F_z_prev	3.62e-04	2.91e-04	7.07e-04	[-0.05, 0.05]
hunt.att.sum.F_z_prev	9.41e-03	9.42e-03	7.38e-03	[-0.05, 0.07]
prop.refuelF_z_prev	-0.01	-0.01	-0.01	[-0.06, 0.04]
prop.succ.dives.F_z_prev	-0.05	-0.05	-0.04	[-0.09, 0.00]
vedba.avg.M_z_prev	-0.12	-0.12	-0.12	[-0.18, -0.06]
hunt.att.sum.M_z_prev	-0.03	-0.03	-0.03	[-0.08, 0.03]
prop.succ.dives.M_z_prev	-0.06	-0.06	-0.06	[-0.11, 0.00]
prop.refuelM_z_prev	-0.06	-0.06	-0.06	[-0.10, -0.02]
n_nest_encounters_z_prev	0.13	0.13	0.13	[0.06, 0.20]
n_out_encounters_z_prev	-0.06	-0.06	-0.06	[-0.11, -0.02]
time_at_nest_M_z_prev	-0.16	-0.16	-0.16	[-0.22, -0.11]
time_at_nest_F_z_prev	-0.16	-0.16	-0.16	[-0.24, -0.08]

Table S8. Female provisioning share on the focal night as response variable (N = 220). All parameters including "_z" or ".z" are continuous variables standardised as z-scores. Parameters including "M" or "F" refer to males and females, respectively, if the letter identifying the sex is missing it means that is relative to both parents combined. Parameters are listed and explained in the STAR*Methods. Predictor names are the same as models above with the addition of "*_prev*" at the end referring to the previous night.

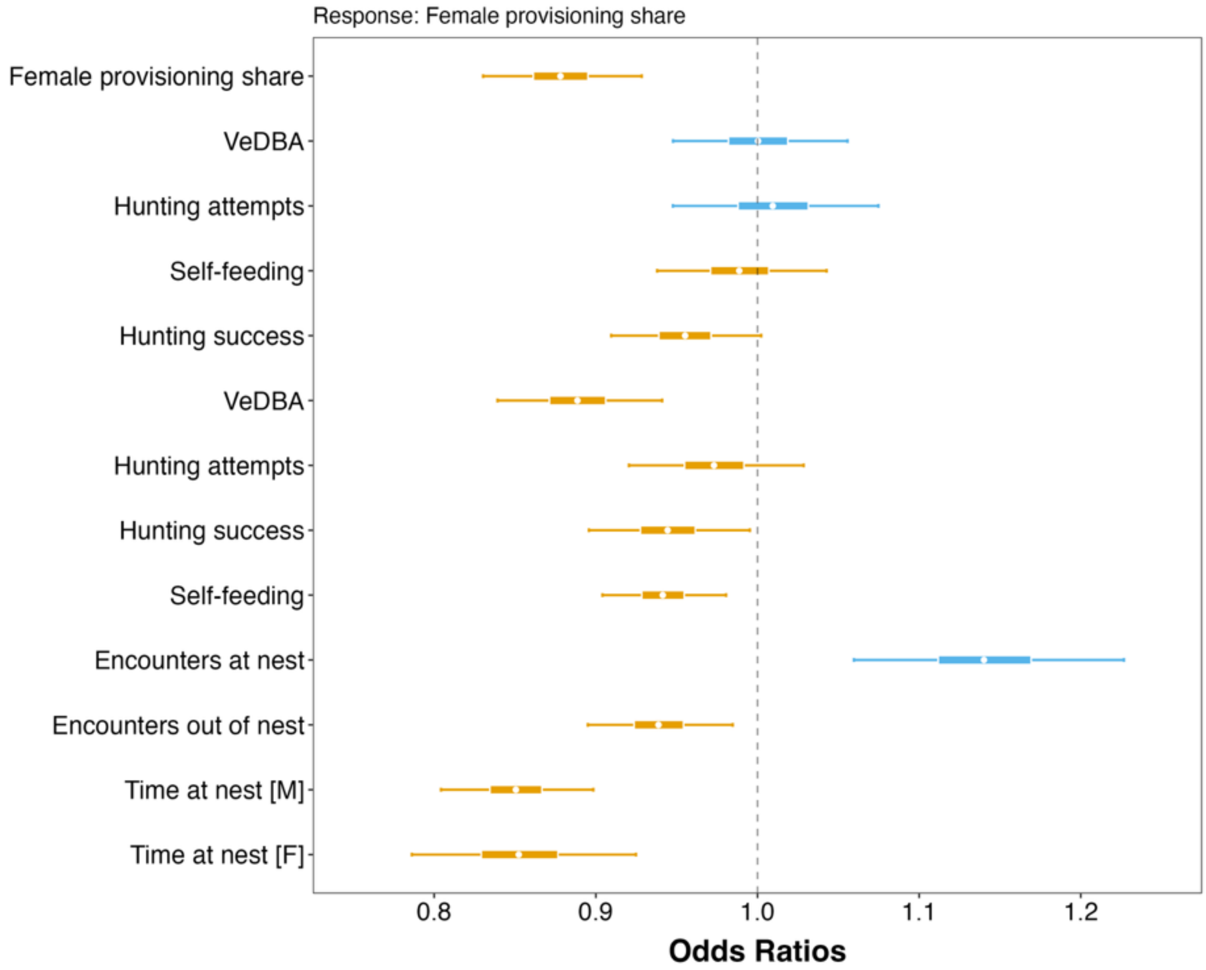

Figure S8. Predictors are referring to the behaviour on the previous night and the response on the focal night. Standardised effects are expressed as posterior distributions (horizontal boxplots) with a mean (white dot), the 50% Credible Intervals (the box) and the 95% Credible Intervals limits (the whiskers).

Parameter	Median	Mean	MAP	95% CI
(Intercept)	2.64	2.64	2.64	[2.43, 2.84]
prey.per.chick_z_prev	-0.46	-0.46	-0.46	[-0.71, -0.22]
prop.bip_z_prev	-0.13	-0.13	-0.12	[-0.37, 0.12]
vedba.avg.F_z_prev	0.10	0.10	0.10	[-0.13, 0.32]
hunt.att.sum.F_z_prev	0.26	0.26	0.26	[0.01, 0.51]
prop.refuelF_z_prev	-0.06	-0.06	-0.06	[-0.26, 0.14]
prop.succ.dives.F_z_prev	0.16	0.16	0.16	[-0.05, 0.37]
vedba.avg.M_z_prev	0.07	0.07	0.07	[-0.15, 0.29]
hunt.att.sum.M_z_prev	0.12	0.12	0.11	[-0.15, 0.38]
prop.succ.dives.M_z_prev	0.08	0.08	0.07	[-0.12, 0.28]
prop.refuelM_z_prev	-0.01	-0.01	-1.78e-03	[-0.19, 0.16]
n_nest_encounters_z_prev	0.23	0.22	0.24	[-0.05, 0.50]
n_out_encounters_z_prev	0.04	0.04	0.03	[-0.12, 0.20]
time_at_nest_M_z_prev	-7.79e-03	-8.22e-03	-3.47e-03	[-0.20, 0.18]
time_at_nest_F_z_prev	-0.14	-0.14	-0.14	[-0.41, 0.14]

Table S9. Prey per nestling by both parents on the focal night as response variable (N = 220). All parameters including "_z" or ".z" are continuous variables standardised as z-scores. Parameters including "M" or "F" refer to males and females, respectively, if the letter identifying the sex is missing it means that is relative to both parents combined. Parameters are listed and explained in the STAR*Methods. Predictor names are the same as models above with the addition of "*_prev*" at the end referring to the previous night.

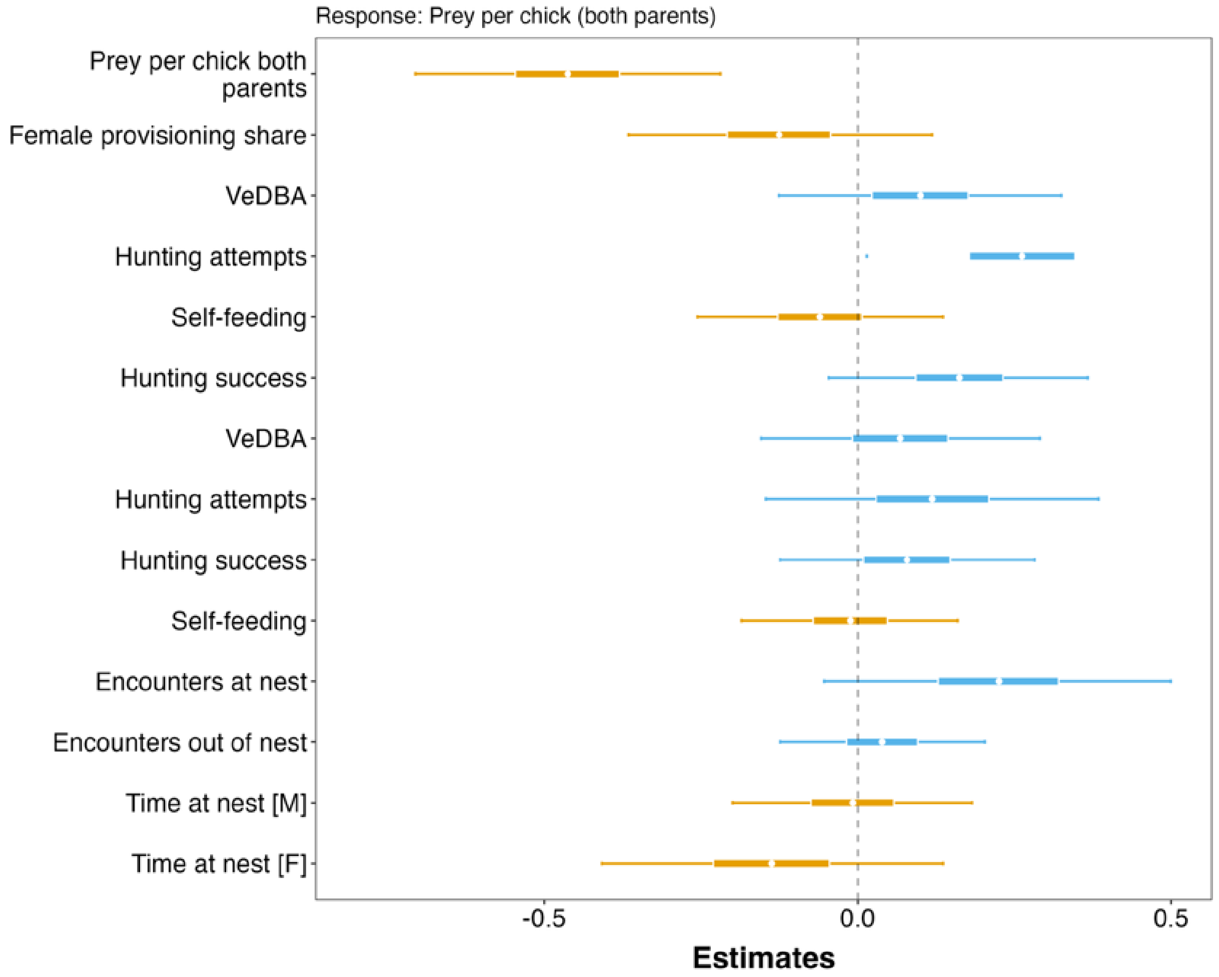

Figure S9. Predictors are referring to the behaviour on the previous night and the response on the focal night. Standardised effects are expressed as posterior distributions (horizontal boxplots) with a mean (white dot), the 50% Credible Intervals (the box) and the 95% Credible Intervals limits (the whiskers).

Parameter	Median	Mean	MAP	95% CI
(Intercept)	0.78	0.78	0.78	[0.67, 0.88]
prey.per.chickF_z_prev	-0.09	-0.09	-0.09	[-0.16, -0.02]
vedba.avg.M_z_prev	-0.03	-0.03	-0.03	[-0.12, 0.06]
hunt.att.sum.M_z_prev	-0.04	-0.04	-0.04	[-0.13, 0.06]
prop.succ.dives.M_z_prev	-0.05	-0.05	-0.05	[-0.13, 0.03]
prop.refuelM_z_prev	0.01	0.01	0.01	[-0.06, 0.08]
time_at_nest_M_z_prev	-0.04	-0.04	-0.04	[-0.13, 0.04]
n_nest_encounters_z_prev	0.03	0.03	0.03	[-0.06, 0.12]
n_out_encounters_z_prev	-0.03	-0.03	-0.02	[-0.10, 0.05]

Table S10. Prey per nestling brought by the mother on the focal night as response variable (N = 220). All parameters including "_z" or ".z" are continuous variables standardised as z-scores. Parameters including "M" or "F" refer to males and females, respectively, if the letter identifying the sex is missing it means that is relative to both parents combined. Parameters are listed and explained in the STAR*Methods. Predictor names are the same as models above with the addition of "*_prev*" at the end referring to the previous night.

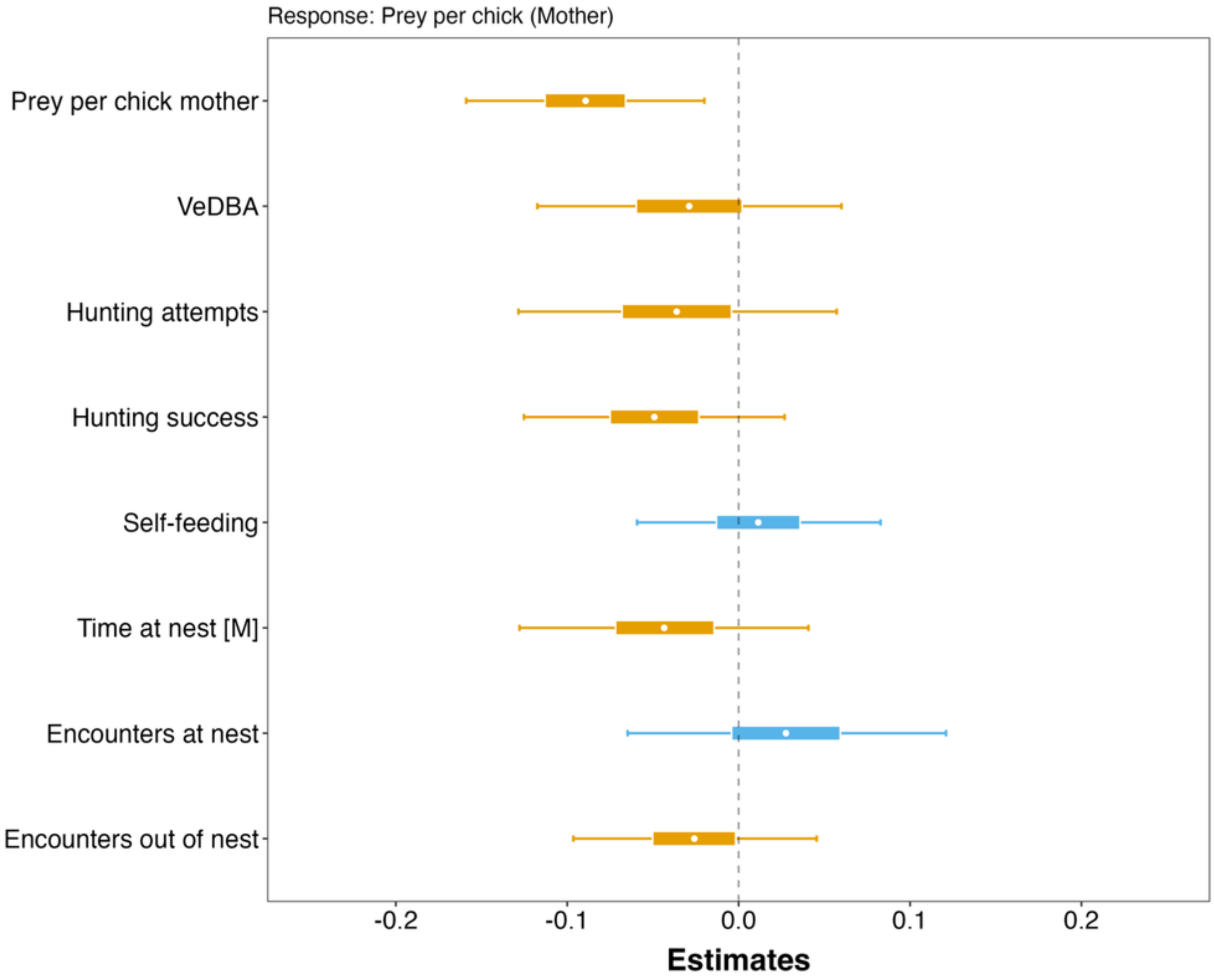

Figure S10. Predictors are referring to the behaviour on the previous night and the response on the focal night. Standardised effects are expressed as posterior distributions (horizontal boxplots) with a mean (white dot), the 50% Credible Intervals (the box) and the 95% Credible Intervals limits (the whiskers).

Parameter	Median	Mean	MAP	95% CI
(Intercept)	1.87	1.87	1.87	[1.68, 2.05]
prey.per.chickM_z_prev	-0.16	-0.16	-0.16	[-0.29, -0.03]
vedba.avg.F_z_prev	-3.61e-03	-3.61e-03	1.06e-03	[-0.18, 0.17]
hunt.att.sum.F_z_prev	0.11	0.11	0.11	[-0.07, 0.28]
prop.succ.dives.F_z_prev	0.08	0.08	0.07	[-0.07, 0.22]
prop.refuelF_z_prev	0.03	0.03	0.04	[-0.12, 0.18]
time_at_nest_F_z_prev	-0.12	-0.12	-0.13	[-0.35, 0.11]
n_nest_encounters_z_prev	0.15	0.15	0.16	[-0.07, 0.37]
n_out_encounters_z_prev	0.06	0.06	0.07	[-0.07, 0.20]

Table S11. Prey per nestling brought by the mother on the focal night as response variable (N = 220). All parameters including "_z" or ".z" are continuous variables standardised as z-scores. Parameters including "M" or "F" refer to males and females, respectively, if the letter identifying the sex is missing it means that is relative to both parents combined. Parameters are listed and explained in the STAR*Methods. Predictor names are the same as models above with the addition of "*_prev*" at the end referring to the previous night.

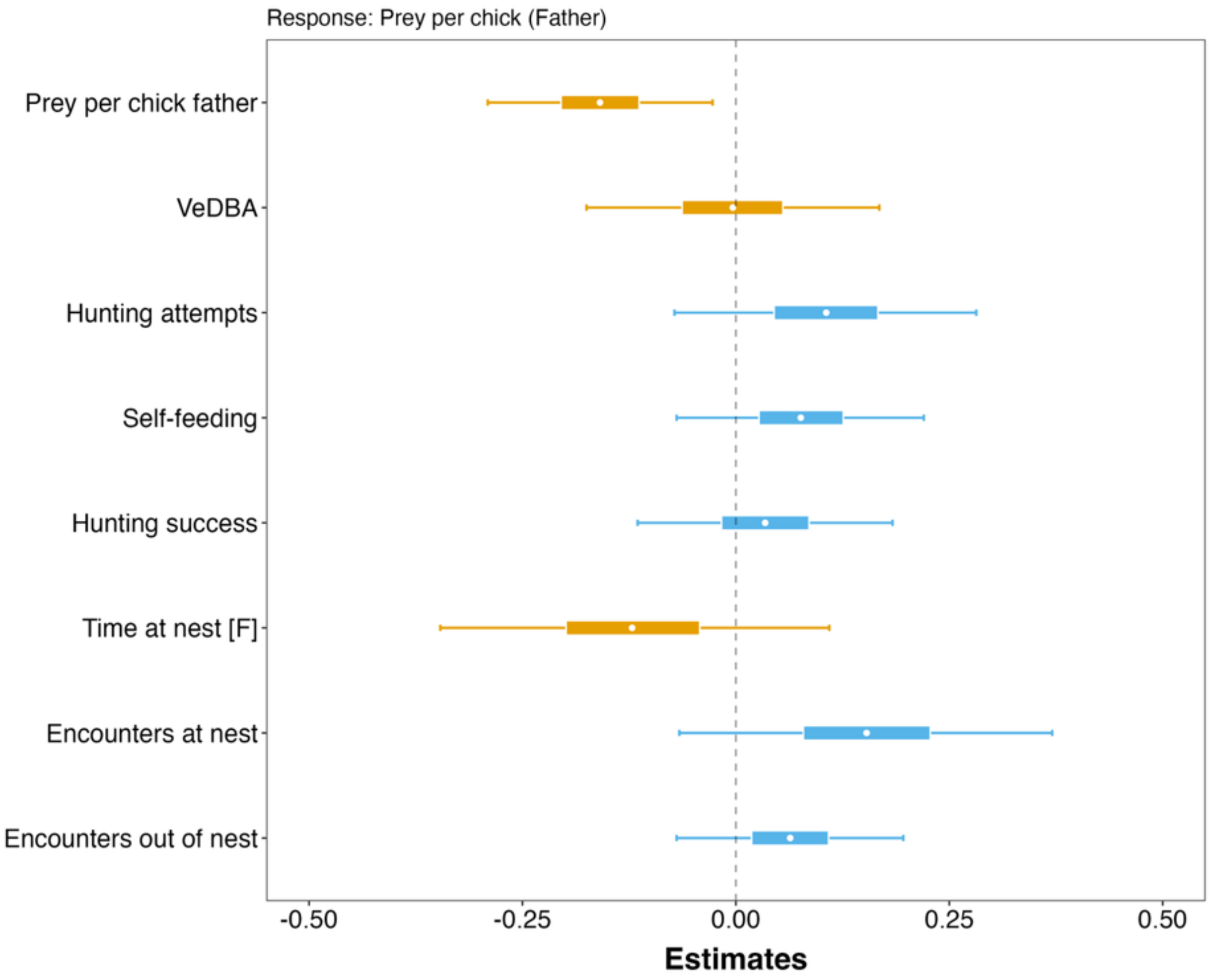

Figure S11. Predictors are referring to the behaviour on the previous night and the response on the focal night. Standardised effects are expressed as posterior distributions (horizontal boxplots) with a mean (white dot), the 50% Credible Intervals (the box) and the 95% Credible Intervals limits (the whiskers).

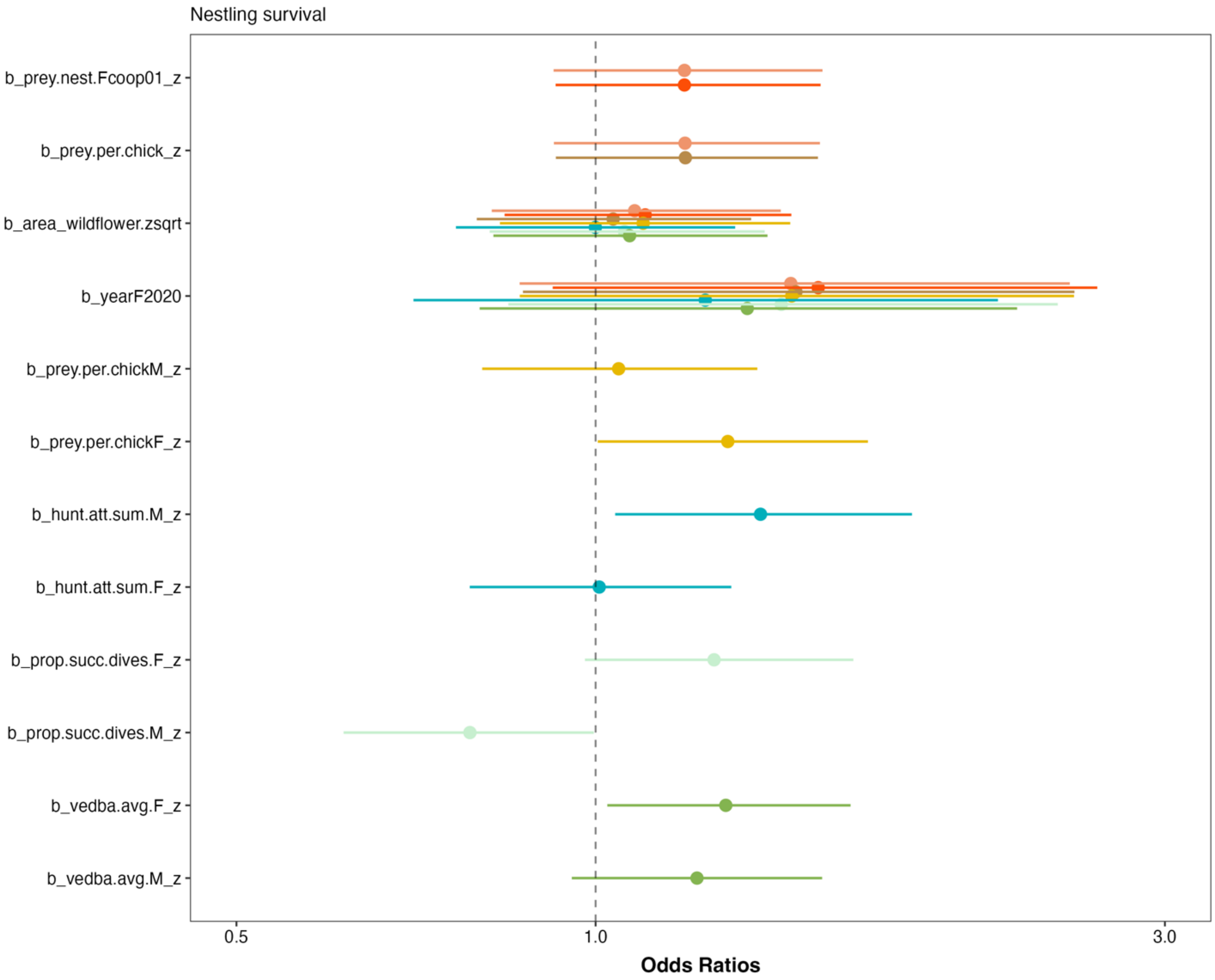

Figure S12. Summary plot of the Bayesian Generalised mixed effect models showing the standardised effects of foraging performance and behaviours of parents, area of wildflower strips, year on nestling survival at tag recovery (nestlings survived divided total eggs laid), N = 68. Standardised effects are expressed as posterior distributions with a mean (dot), and the 95% Credible Intervals limits (line). Different colours highlight different models (see Table S19). All parameters including "_z" or ".z" are continuous variables standardised as z-scores. Parameters including "M" or "F" refer to males and females, respectively, if the letter identifying the sex is missing it means that is relative to both parents combined. Parameters are listed and explained in the STAR*Methods.

	Parameter	Median	Mean	MAP	95% CrI
Model 1					
	(Intercept)	0.81	0.81	0.81	[0.50, 1.16]
	prop.bip_z	0.17	0.17	0.17	[-0.08, 0.44]
	prey.per.chick_z	0.17	0.17	0.16	[-0.08, 0.44]
	area_wildflower.zsqrt	0.07	0.08	0.08	[-0.20, 0.36]
	yearF2020	0.38	0.38	0.39	[-0.15, 0.92]
	Random effects SD/Cor: BroodID	0.39	0.39	0.45	[0.02, 0.85]
Model 2					
	(Intercept)	0.77	0.78	0.77	[0.47, 1.12]
	prop.bip_z	0.17	0.17	0.18	[-0.08, 0.43]
	area_wildflower.zsqrt	0.10	0.10	0.09	[-0.18, 0.38]
	yearF2020	0.43	0.43	0.42	[-0.09, 0.97]
	Random effects SD/Cor: BroodID	0.40	0.40	0.41	[0.03, 0.85]
Model 3					
	(Intercept)	0.79	0.80	0.79	[0.49, 1.14]
	prey.per.chick_z	0.18	0.18	0.18	[-0.08, 0.43]
	area_wildflower.zsqrt	0.03	0.03	0.05	[-0.23, 0.30]
	yearF2020	0.39	0.39	0.39	[-0.14, 0.92]
	Random effects SD/Cor: BroodID	0.37	0.38	0.40	[0.02, 0.84]
Model 4					
	(Intercept)	0.81	0.81	0.81	[0.50, 1.16]
	prey.per.chickM_z	0.04	0.04	0.05	[-0.22, 0.31]
	prey.per.chickF_z	0.26	0.26	0.25	[0.01, 0.52]
	area_wildflower.zsqrt	0.09	0.09	0.09	[-0.18, 0.38]
	yearF2020	0.38	0.38	0.37	[-0.15, 0.92]
	Random effects SD/Cor: BroodID	0.38	0.39	0.39	[0.02, 0.84]
Model 5					
	(Intercept)	0.87	0.88	0.87	[0.56, 1.23]
	hunt.att.sum.M_z	0.32	0.32	0.31	[0.04, 0.61]
	hunt.att.sum.F_z	8.22e-03	8.42e-03	0.02	[-0.24, 0.26]
	area_wildflower.zsqrt	-5.78e-04	9.93e-05	-7.98e-03	[-0.27, 0.27]
	yearF2020	0.21	0.21	0.21	[-0.33, 0.76]
	Random effects SD/Cor: BroodID	0.35	0.36	0.32	[0.02, 0.82]
Model 6					
	(Intercept)	0.82	0.83	0.83	[0.52, 1.17]
	prop.succ.dives.F_z	0.23	0.23	0.23	[-0.02, 0.50]
	prop.succ.dives.M_z	-0.24	-0.24	-0.25	[-0.49, -0.01]
	area_wildflower.zsqrt	0.05	0.06	0.05	[-0.21, 0.33]
	yearF2020	0.36	0.36	0.34	[-0.17, 0.89]
	Random effects SD/Cor: BroodID	0.34	0.36	0.37	[0.02, 0.81]
Model 7					
	(Intercept)	0.84	0.85	0.83	[0.54, 1.19]
	vedba.avg.F_z	0.25	0.25	0.26	[0.02, 0.49]
	vedba.avg.M_z	0.20	0.20	0.21	[-0.04, 0.44]
	area_wildflower.zsqrt	0.06	0.06	0.06	[-0.19, 0.33]
	yearF2020	0.29	0.29	0.30	[-0.22, 0.81]
	Random effects SD/Cor: BroodID	0.31	0.33	0.32	[0.02, 0.77]

Table S12. Summary of models predicting nestling survival (N = 68, see Figure S12). All parameters including "_z" or ".z" are continuous variables standardised as z-scores. Parameters including "M" or "F" refer to males and females, respectively, if the letter identifying the sex is missing it means that is relative to both parents combined. Parameters are listed and explained in the STAR*Methods.

Parameter	Median	Mean	MAP	95% CrI
(Intercept)	256.30	256.28	256.36	[246.81, 265.64]
prop.bip_z	5.79	5.78	5.67	[0.00, 11.50]
time.group2	74.76	74.78	74.66	[67.49, 82.09]
time.group3	109.68	109.71	109.76	[102.08, 117.43]
Rank2	-8.96	-8.97	-8.77	[-16.22, -1.85]
Rank3	-13.32	-13.31	-13.75	[-21.22, -5.52]
Rank4	-45.27	-45.30	-45.10	[-55.63, -35.11]

Table S13. Summary of posterior distribution (model without interactions), N=1045, response variable is nestling's absolute weight. Parameters are listed and explained in the STAR*Methods.

Parameter	Median	Mean	MAP	95% CrI
(Intercept)	256.11	256.08	256.27	[246.57, 265.56]
prop.bip_z	5.72	5.68	5.75	[-7.65, 18.82]
time.group2	74.53	74.52	74.76	[67.21, 81.83]
time.group3	110.10	110.12	109.98	[102.35, 117.93]
Rank2	-9.01	-9.01	-8.85	[-16.26, -1.77]
Rank3	-12.41	-12.41	-12.72	[-20.47, -4.47]
Rank4	-46.45	-46.51	-46.40	[-57.13, -36.28]
prop.bip_z:time.group2	-1.17	-1.15	-1.10	[-15.65, 13.47]
prop.bip_z:time.group3	-3.35	-3.29	-3.69	[-17.72, 11.31]
prop.bip_z:Rank2	-0.11	-0.07	-0.30	[-15.71, 15.98]
prop.bip_z:Rank3	-0.57	-0.45	-0.93	[-17.64, 17.10]
prop.bip_z:Rank4	-1.03	-0.93	-1.71	[-19.11, 17.64]
prop.bip_z:time.group2:Rank2	5.47	5.45	5.58	[-14.17, 24.93]
prop.bip_z:time.group3:Rank2	5.28	5.23	5.46	[-13.50, 23.68]
prop.bip_z:time.group2:Rank3	-0.77	-0.80	-0.63	[-21.74, 20.23]
prop.bip_z:time.group3:Rank3	-5.49	-5.59	-5.70	[-26.02, 14.49]
prop.bip_z:time.group2:Rank4	11.57	11.53	12.10	[-9.71, 32.92]
prop.bip_z:time.group3:Rank4	10.08	9.97	10.58	[-12.15, 31.61]

Table S14. Summary of posterior distribution (model including interactions), N=1045, response variable is nestling's absolute weight. Parameters are listed and explained in the STAR*Methods.

time.group	Rank	Fcoop trend	lower.HPD	upper.HPD
1	1	5.72	-7.834	18.6
2	1	4.54	-5.847	14.8
3	1	2.42	-6.701	11.3
1	2	5.65	-6.094	17.1
2	2	9.92	-0.226	20.0
3	2	7.60	-1.342	16.4
1	3	5.23	-7.652	18.6
2	3	3.27	-7.119	13.9
3	3	-3.66	-12.686	5.0
1	4	4.75	-9.012	18.4
**2**	**4**	**15.15**	**4.347**	**26.4**
**3**	**4**	**11.44**	**2.288**	**20.7**

Table S15. Summary of posterior slopes of third-way interaction, N=1045, response variable is nestling's absolute weight. Parameters are listed and explained in the STAR*Methods. Point estimate displayed: median; HPD interval probability: 0.95; meaningful slopes in bold.

Parameter	Median	Mean	MAP	95% CrI
(Intercept)	109.78	109.76	110.14	[103.91, 115.54]
prop.bip_z	3.22	3.20	3.43	[-1.61, 7.98]
time.group2	56.43	56.42	56.45	[54.58, 58.29]
time.group3	164.61	164.61	164.59	[161.76, 167.42]
Rank2	-14.09	-14.09	-13.92	[-18.67, -9.47]
Rank3	-27.87	-27.86	-28.03	[-32.61, -23.16]
Rank4	-56.80	-56.79	-57.07	[-61.23, -52.34]

Table S16. Summary of posterior distribution (model without interactions), N=1045, response variable is nestling's wing length. Parameters are listed and explained in the STAR*Methods.

Parameter	Median	Mean	MAP	95% CrI
(Intercept)	109.70	109.70	109.72	[103.96, 115.47]
prop.bip_z	2.64	2.62	2.77	[-3.50, 8.74]
time.group2	56.43	56.42	56.55	[54.58, 58.26]
time.group3	164.41	164.42	164.39	[161.57, 167.29]
Rank2	-13.89	-13.89	-14.00	[-18.54, -9.26]
Rank3	-27.80	-27.81	-27.66	[-32.39, -23.17]
Rank4	-57.33	-57.32	-57.49	[-61.75, -52.85]
prop.bip_z:time.group2	-2.71	-2.72	-2.55	[-6.98, 1.50]
prop.bip_z:time.group3	-3.72	-3.73	-3.93	[-10.62, 3.16]
prop.bip_z:Rank2	1.68	1.70	1.49	[-3.88, 7.31]
prop.bip_z:Rank3	-0.05	-0.05	0.17	[-5.78, 5.69]
prop.bip_z:Rank4	3.51	3.52	3.39	[-1.96, 9.06]
prop.bip_z:time.group2:Rank2	-0.18	-0.17	-0.34	[-5.81, 5.51]
prop.bip_z:time.group3:Rank2	5.49	5.49	5.57	[-3.43, 14.48]
prop.bip_z:time.group2:Rank3	0.45	0.44	0.39	[-5.33, 6.07]
prop.bip_z:time.group3:Rank3	3.17	3.15	3.80	[-5.71, 12.15]
prop.bip_z:time.group2:Rank4	3.14	3.13	3.11	[-2.30, 8.59]
prop.bip_z:time.group3:Rank4	5.81	5.84	5.18	[-2.69, 14.45]

Table S17. Summary of posterior distribution (model with interactions), N=1045, response variable is nestling's wing length. Parameters are listed and explained in the STAR*Methods.

time.group	Rank	Fcoop trend	lower.HPD	upper.HPD
1	1	2.638	-3.523	8.70
2	1	-0.100	-6.041	5.85
3	1	-1.114	-8.960	7.15
1	2	4.319	-1.457	10.11
2	2	1.445	-4.394	7.22
3	2	6.082	-1.391	13.43
1	3	2.569	-3.429	8.57
2	3	0.296	-5.367	6.15
3	3	1.988	-5.187	9.26
**1**	**4**	**6.169**	**0.260**	**11.64**
**2**	**4**	**6.566**	**0.909**	**12.01**
**3**	**4**	**8.259**	**1.632**	**15.12**

Table S18. Summary of posterior slopes of third-way interaction, N=1045, response variable is nestling's wing length. Parameters are listed and explained in the STAR*Methods. Point estimate displayed: median; HPD interval probability: 0.95; meaningful slopes in bold.

Parameter	Median	Mean	MAP	95% CI
(Intercept)	5.35	5.35	5.36	[4.51, 6.20]
prop.bip_z	0.76	0.76	0.73	[-0.57, 2.10]
time.group3	-5.93	-5.93	-5.96	[-6.67, -5.20]
Rank2	0.73	0.73	0.75	[-0.12, 1.58]
Rank3	1.51	1.51	1.57	[0.60, 2.41]
Rank4	2.94	2.94	2.93	[2.13, 3.73]

Table S19. Summary of posterior distribution (model without interactions), N=726, response variable is nestling's daily weight change. Parameters are listed and explained in the STAR*Methods.

Parameter	Median	Mean	MAP	95% CrI
sigma_Intercept	1.67	1.67	1.67	[1.55, 1.80]
(Intercept)	5.35	5.35	5.34	[4.51, 6.21]
prop.bip_z	0.77	0.77	0.77	[-0.60, 2.11]
time.group3	-5.93	-5.93	-5.91	[-6.67, -5.19]
Rank2	0.73	0.73	0.76	[-0.12, 1.57]
Rank3	1.50	1.51	1.48	[0.60, 2.41]
Rank4	2.94	2.94	2.93	[2.12, 3.75]
prop.bip_z:time.group3	-1.17	-1.17	-1.17	[-2.67, 0.33]
prop.bip_z:Rank2	-0.50	-0.50	-0.36	[-2.39, 1.40]
prop.bip_z:Rank3	-0.49	-0.49	-0.37	[-2.54, 1.59]
prop.bip_z:Rank4	0.05	0.05	0.02	[-1.76, 1.85]
prop.bip_z:time.group3:Rank2	1.04	1.05	1.03	[-1.11, 3.20]
prop.bip_z:time.group3:Rank3	0.30	0.30	0.26	[-2.03, 2.61]
prop.bip_z:time.group3:Rank4	-0.39	-0.39	-0.35	[-2.43, 1.63]

Table S20. Summary of posterior distribution (model with interactions) , N=726, response variable is nestling's daily weight change. Parameters are listed and explained in the STAR*Methods.

time.group	Rank	Fcoop trend	lower.HPD	upper.HPD
2	1	0.770	-0.572	2.130
3	1	-0.402	-1.131	0.318
2	2	0.262	-1.140	1.646
3	2	0.142	-0.615	0.914
2	3	0.275	-1.314	1.889
3	3	-0.592	-1.399	0.239
2	4	0.818	-0.409	2.047
**3**	**4**	**-0.748**	**-1.386**	**-0.106**

Table S21. Summary of posterior slopes of third-way interaction, N=726, response variable is nestling's daily weight change. Parameters are listed and explained in the STAR*Methods. Point estimate displayed: median; HPD interval probability: 0.95; meaningful slopes in bold.

Parameter	Median	Mean	MAP	95% CI
(Intercept)	5.06	5.06	5.06	[4.82, 5.30]
prop.bip_z	-0.16	-0.16	-0.17	[-0.53, 0.21]
time.group3	-1.33	-1.33	-1.33	[-1.49, -1.16]
Rank2	0.26	0.26	0.25	[-0.01, 0.53]
Rank3	0.51	0.51	0.52	[0.27, 0.76]
Rank4	0.65	0.65	0.65	[0.40, 0.90]

Table S22. Summary of posterior distribution (model without interactions), N=726, response variable is nestling's daily wing length growth. Parameters are listed and explained in the STAR*Methods.

Parameter	Median	Mean	MAP	95% CI
sigma_Intercept	0.40	0.40	0.40	[0.25, 0.55]
(Intercept)	5.06	5.06	5.05	[4.82, 5.30]
prop.bip_z	-0.16	-0.16	-0.16	[-0.53, 0.21]
time.group3	-1.33	-1.33	-1.32	[-1.49, -1.16]
Rank2	0.26	0.26	0.27	[-0.01, 0.53]
Rank3	0.51	0.51	0.53	[0.27, 0.76]
Rank4	0.65	0.65	0.65	[0.40, 0.90]
prop.bip_z:time.group3	0.02	0.02	0.04	[-0.42, 0.47]
prop.bip_z:Rank2	8.40e-03	9.53e-03	-2.70e-03	[-0.47, 0.48]
prop.bip_z:Rank3	0.15	0.15	0.17	[-0.28, 0.58]
prop.bip_z:Rank4	0.38	0.38	0.38	[-0.06, 0.81]
prop.bip_z:time.group3:Rank2	0.13	0.13	0.13	[-0.46, 0.71]
prop.bip_z:time.group3:Rank3	-0.08	-0.09	-0.09	[-0.61, 0.44]
prop.bip_z:time.group3:Rank4	-0.37	-0.37	-0.37	[-0.90, 0.16]

Table S23. Summary of posterior distribution (model with interactions), N=726, response variable is nestling's daily wing length growth. Parameters are listed and explained in the STAR*Methods.

time.group	Rank	prop.bip_z.trend	lower.HPD	upper.HPD
2	1	-0.15810	-0.5259	0.2109
3	1	-0.13367	-0.3999	0.1388
2	2	-0.14889	-0.4625	0.1718
3	2	0.00209	-0.2310	0.2343
2	3	-0.00560	-0.2479	0.2249
3	3	-0.06713	-0.2351	0.0994
**2**	**4**	**0.22104**	**-0.0103**	**0.4532**
**3**	**4**	**-0.12406**	**-0.2952**	**0.0348**

Table S24. Summary of posterior slopes of third-way interaction, N=726, response variable is nestling's daily wing length growth. Parameters are listed and explained in the STAR*Methods.. Point estimate displayed: median; HPD interval probability: 0.95; meaningful slopes in bold.
